# Synthetic Routes to Coumarin(Benzopyrone)-Fused Five-Membered Aromatic Heterocycles Built on the α-Pyrone Moiety. Part II: Five-Membered Aromatic Rings with Multi Heteroatoms

**DOI:** 10.3390/molecules26113409

**Published:** 2021-06-04

**Authors:** Eslam Reda El-Sawy, Ahmed Bakr Abdelwahab, Gilbert Kirsch

**Affiliations:** 1 National Research Centre, Chemistry of Natural Compounds Department, Dokki, Cairo 12622, Egypt; eslamelsawy@gmail.com; 2 Plant Advanced Technologies (PAT), 54500 Vandoeuvre-les-Nancy, France; ahm@plantadvanced.com; 3 Laboratoire Lorrain de Chimie Moléculaire (L.2.C.M.), Université de Lorraine, 57078 Metz, France

**Keywords:** coumarins, benzopyrones, pyrazole, imidazole, thiazole, oxazole, triazole, thiadiazole

## Abstract

Coumarins are natural heterocycles that widely contribute to the design of various biologically active compounds. Fusing different aromatic heterocycles with coumarin at its 3,4-position is one of the interesting approaches to generating novel molecules with various biological activities. During our continuing interest in assembling information about fused five-membered aromatic heterocycles, and after having presented mono-hetero-atomic five-membered aromatic heterocycles in Part I. The current review Part II is intended to present an overview of the different synthetic routes to coumarin (benzopyrone)-fused five-membered aromatic heterocycles with multi-heteroatoms built on the pyrone ring, covering the literature from 1945 to 2021.

## 1. Introduction

The fusion of the pyrone ring with the benzene nucleus gives rise to a class of heterocyclic compounds known as benzopyrone [1]. Coumarin is one of the benzopyrones (1,2-benzopyrones or 2H-[1] benzopyran-2-ones) and represents an important family of oxygen-containing heterocycles widely distributed in nature [1]. The incorporation of another heterocyclic moiety into coumarin enriches the properties of the parent structure and the resulting compounds may exhibit promising properties [2,3,4,5]. Certain derivatives of 3,4-heterocycle-fused coumarins play an important role in medicinal chemistry and have been extensively used as versatile building blocks in organic synthesis [2,3,5,6,7]. Many examples of biologically active coumarins containing 3,4-heterocycles-fused were cited in the literature [2,3,8] including antimicrobial [9,10,11,12], antiviral [13,14], anticancer [15,16,17], antioxidant [18,19], and anti-inflammatory [20,21] activities.

The development of synthetic pathways towards active coumarins containing heterocyclics has attracted great interest from researchers [5]. Significant efforts have been focused on developing new methodologies to enrich structural libraries and reduce the number of synthetic steps of novel coumarin derivatives [5,22].

In proceeding to our interest in coumarin(benzopyrone)-fused five-membered aromatic heterocycles built on the α-pyrone ring, which was recently issued in Part I [22]. The present review, Part II, describes the components which have multi heteroatom in an aromatic fused ring with the pyrone part of coumarin. The synthetic pathways of the investigated scaffolds provided systems containing oxygen, nitrogen, and sulfur in their core structure.

## 2. Synthesis of Benzopyrone-Fused Five-Membered Aromatic Heterocycles

### 2.1. Five-Membered Aromatic Rings with Two Heteroatoms

#### 2.1.1. Two Identical Heteroatoms (N-N)

##### Pyrazole

**Figure 1:**
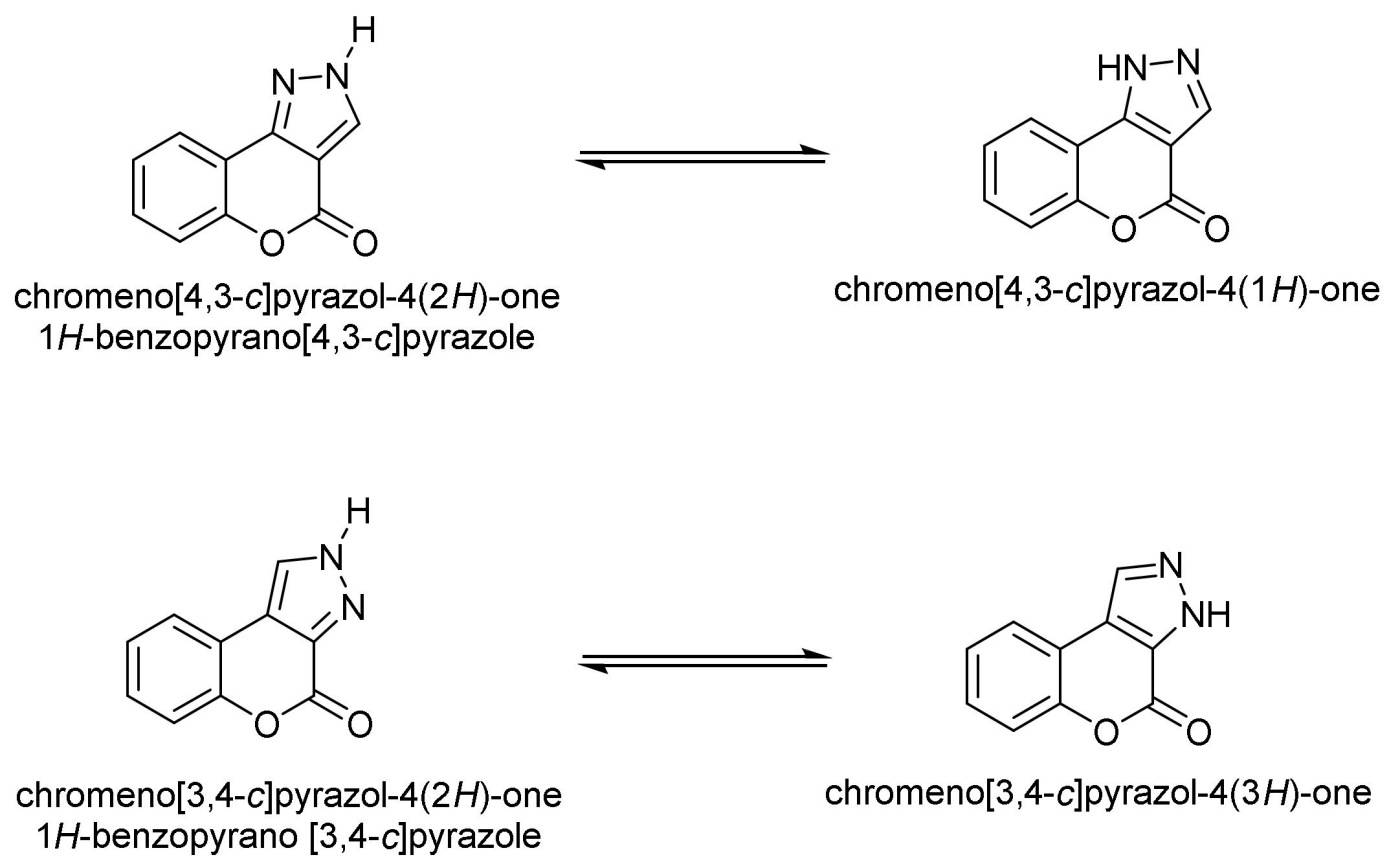
The isomers of fused chromeno-pyrazole.

Fusion of pyrazole ring with the pyrone ring of coumarin results in formation of two structural isomers, namely chromeno[4,3-*c*]pyrazol-4(2*H*)-one, chromeno[4,3-*c*]pyrazol-4(1*H*)-one, (1*H*-benzopyrano[4,3-*c*]pyrazole), and chromeno[3,4-*c*]pyrazol-4(2*H*)-one, chromeno[3,4-*c*]pyrazol-4(3*H*)-one, (1*H*-benzopyrano[3,4-*c*]pyrazole) (Figure 1).

Chromeno[4,3-c]pyrazol-4(2*H*)-one; (1*H*-Benzopyrano[4,3-c]pyrazole)

Many synthetic protocols reported the synthesis of chromeno[4,3-*c*]pyrazol-4(2*H*)-one including the pyrazole and/or the pyrone-ring construction. The synthesis of the pyrazole ring in the literature started from coumarin, 4-hydroxy, 3-aldehyde, or 3-acetyl coumarin in addition to the chromone derivatives.

Pyrazole Construction

**Scheme 1:**
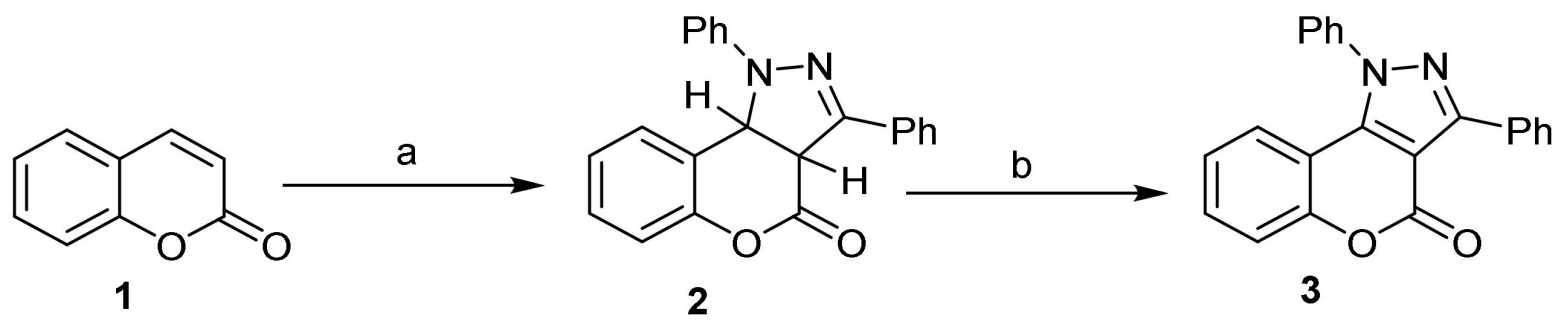
Cycloaddition of diphenylnitrilimine to coumarin. Reagents and conditions. (**a**) diphenylnitrilimine (DPNI), benzene, TEA, compound **2**, 65% yield, compound **5**, 65% yield; (**b**) lead tetraacetate, dichloromethane, r.t., 12 h, 83% yield.

Shawali and his co-workers described the 1,3-dipolar additions of diphenylnitrilimine (DPNI) to coumarin (**1**) to afford 1,3-diphenyl-3a,9b-dihydro-4-oxo-1*H*-chromeno[4,3-*c*]pyrazol-4(1*H*)one (**2**) [23]. Upon dehydrogenation of 2 with lead tetra-acetate the corresponding 1,3-diphenyl-chromeno[4,3-c]pyrazol-4(1H)one (3) was obtained (Scheme 1).

**Scheme 2:**
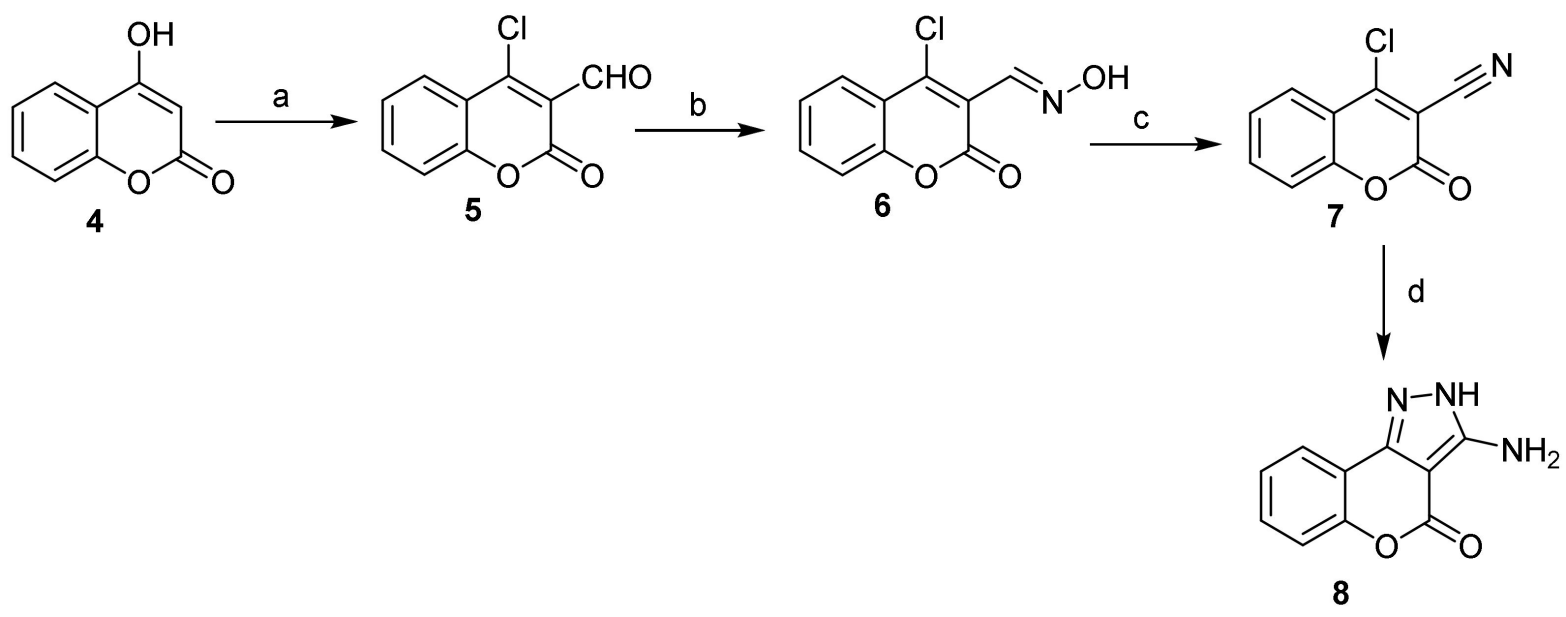
Synthesis of 3-aminochromeno[4,3-*c*]pyrazol-4(1*H*)one (**8**). Reagents and conditions. (**a**) POCl_3_, DMF, CHCl_3_; (**b**) NH_2_OH. HCl, AcONa, EtOH; (**c**) POCl_3_; (**d**) N_2_H_4_. H_2_O, EtOH, 80% yield.

3-Aminochromeno[4,3-*c*]pyrazol-4(1*H*)one (**8**) was prepared through multi-step reactions starting from 4-hydroxycoumarin (**4**) [24]. Vilsmeier–Haack formylation of **4** developed 4-chlorocoumarin-3-carboxaldehyde (**5**). The reaction of **5** with hydroxylamine hydrochloride followed with phosphorus oxychloride gave the corresponding 4-chlorocoumarin-3-carbonitrile (**7**). Compound **7** under the reaction with hydrazine hydrate provided the target compound, 3-aminochromeno[4,3-*c*]pyrazol-4(1*H*)one (**8**) (Scheme 2).

**Scheme 3:**
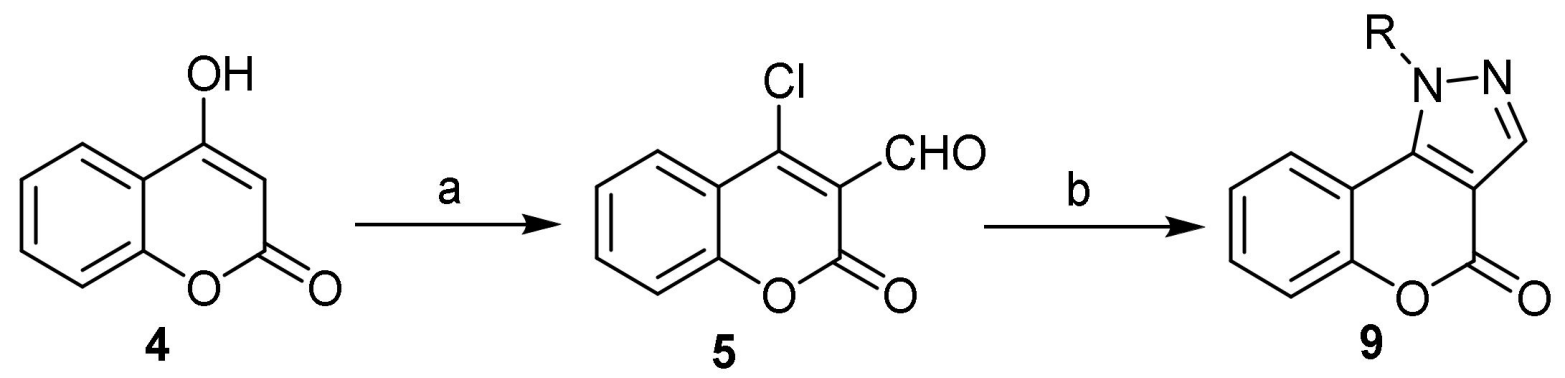
Synthesis of chromeno[4,3-*c*]pyrazol-4(1*H*)ones **9**. Reagents and conditions. (**a**) POCl_3_, DMF, CHCl_3_; (**b**) NH_2_NHR, EtOH, TEA or NaOAc, 25 °C, 2 h, five outputs with 67–89% yield [18], 14 outputs with 38–69% yield [25].

**Scheme 4:**
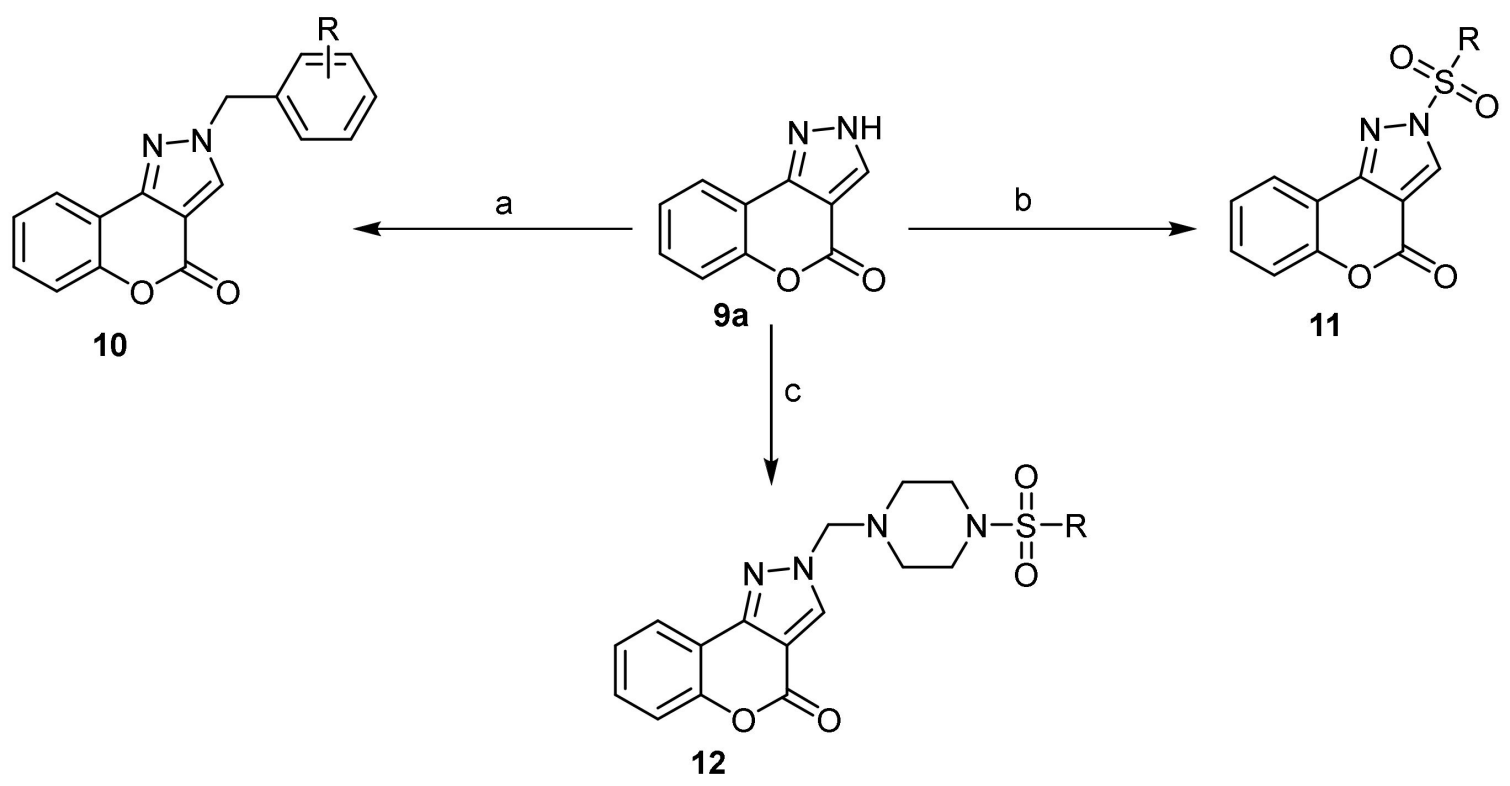
Synthesis of 2-substituted-1*H*-chromeno[4,3-*c*]pyrazol-4(1*H*)ones **10–12**. Reagents and conditions. (**a**) Benzyl bromides, DMF, Cs_2_CO_3_, 100 °C, 10–12 h, 13 outputs; (**b**) benzene(alkyl)sulfonyl chlorides, DCM, TEA, 0 °C, 6–8 h, 28 outputs with 33–52% yield; (**c**) *N*-alkyl sulfonyl piperazines, HCHO, EtOH, rt, 4–6 h, 26 outputs with 37–65% yield.

The preparation of chromen[4,3-*c*]pyrazol-4-ones **9** from the reaction of 3-formyl-4-chlorocoumarin (**5**) with the appropriate aryl or alkyl hydrazine hydrochloride in the presence of base was intensively investigated (Scheme 3) [10,18,19,25,26,27,28,29,30]. Compound **9a** was employed as starting material to enrich the derivatives of chromen[4,3-*c*]pyrazol-4-ones **10–12** through the reaction with benzyl bromides [26], alkyl sulfonyl chlorides [28], or *N*-piperazine sulfonyl chlorides [30] (Scheme 4).

**Scheme 5:**
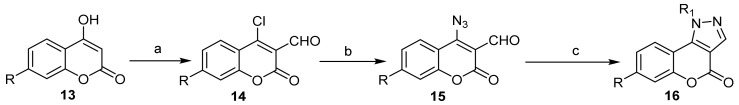
Synthesis of chromeno[4,3-*c*]pyrazol-4(2*H*)-ones **16**. Reagents and conditions. (**a**) POCl_3_, DMF, CHCl_3_, 4 outputs with 65–80% yield; (**b**) NaN_3_, DMF, 4 outputs with 50–80% yield; (**c**) R_1_NHNH_2,_ DMF, 40–50 °C, six outputs with 70–90% yield.

Steinfiihrer et al., synthesized chromeno[4,3-*c*]pyrazol-4(2*H*)-ones **16** in a three-step reaction starting from 4-hydroxycoumarin derivatives **13**. The intermediate, 4-azido-3-coumarincarboxaldehydes **15** was produced in situ from 4-chlorocoumarin-3-carboxaldehydes **14** which subsequently reacted with some hydrazine derivatives to deliver the final products (Scheme 5) [31].

**Scheme 6:**
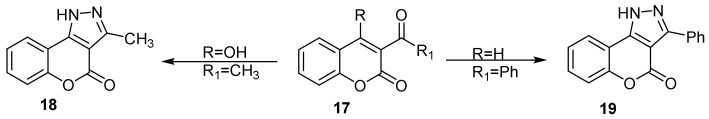
3-Coumarinyl alkyl ketone in the synthesis of chromeno[4,3-*c*]pyrazol-4(2*H*)-ones **18** and **19**. Reagents and conditions. NH_2_NH_2_.H_2_O, EtOH, TEA, **18**: 75% yield, **19**: 58% yield.

Additionally, 3-coumarinyl alkyl ketones, such as 4-hydroxy-3-acetylcoumarin (**17a**) [32], and 3-coumarinyl phenyl ketone (**17b**) [12] were the starting materials in the preparation of chromeno[4,3-*c*]pyrazol-4(2*H*)-ones **18** and **19** by the base-catalyzed reaction with hydrazine hydrate (Scheme 6).

**Scheme 7:**
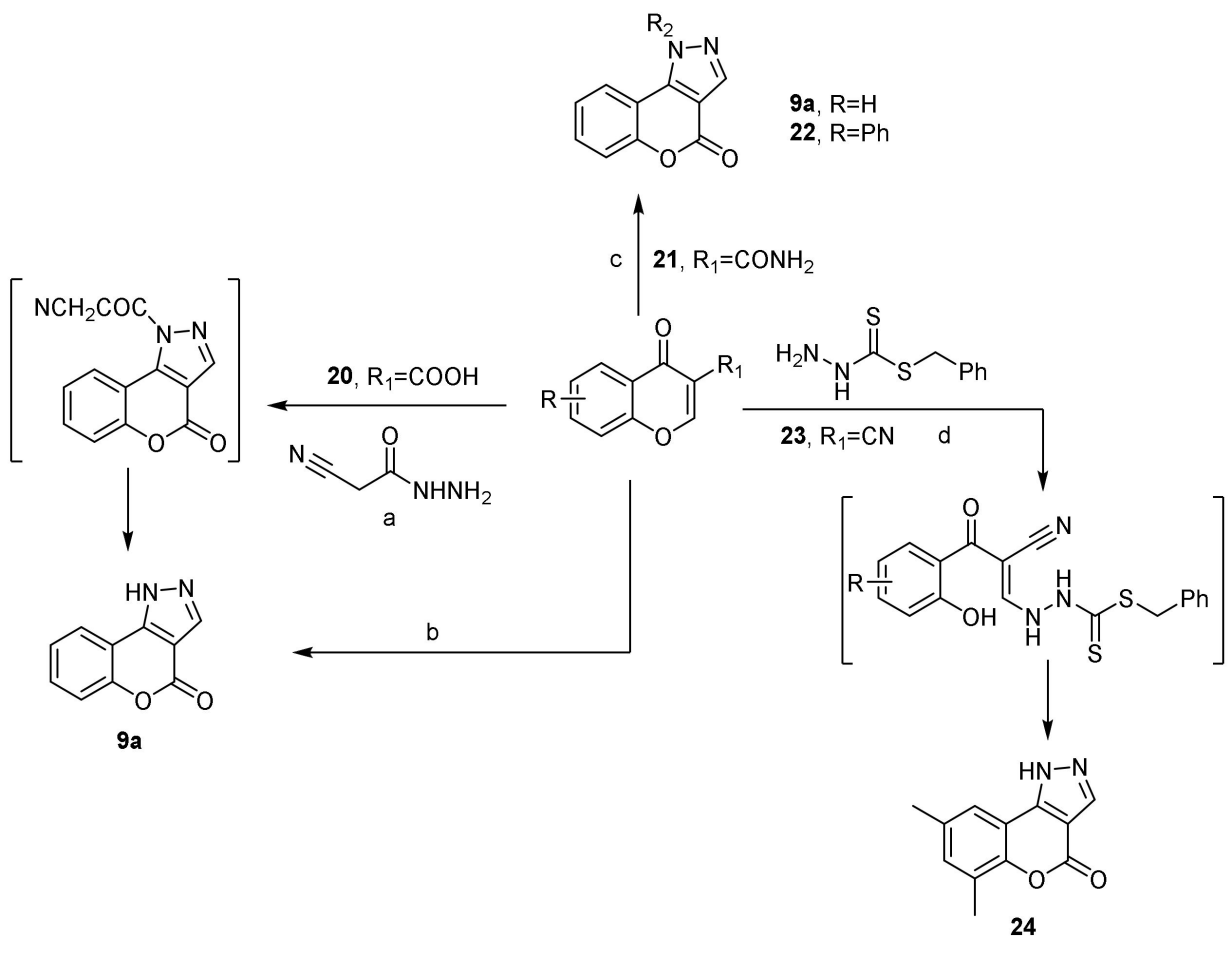
Synthesis of chromeno[4,3-*c*]pyrazol-4(2*H*)-ones **9a**, **22**, and **24**. Reagents and conditions. (**a**) AcOH, 2 h, reflux, 48% yield; (**b**) NH_2_NH_2_. H_2_O, AcOH, 2 h, reflux, 48% yield; (**c**) NH_2_NH_2_. H_2_O, or NH_2_NHPh, EtOH, 2 h, reflux, R=H: 56%, R=Ph: 46% yield; (**d**) DMF, 30 min, reflux, 63% yield.

The transformation of three-substituted chromone to chromeno[4,3-*c*]pyrazol-4(2*H*)-ones was within the scope of interest of Ibrahim’s research group [33,34,35,36]. In 2008, they studied the ring transformation of chromone-3-carboxylic acid (**20**) under the reaction with 2-cyanoacetohydrazide or the hydrazine hydrate as the nucleophile to give the corresponding chromeno[4,3-*c*]pyrazol-4(2*H*)-one (**9a**) (Scheme 7) [33]. Furthermore, they examined the chemical reactivity of chromone-3-carboxamide (**21**) towards hydrazine hydrate or phenylhydrazine and they could construct the pyrazole within the scaffold (Scheme 7) [34]. Furthermore, 3-cyano-2,6-dimethyl chromone (**23**) was allowed to react with the nucleophile *S*-benzyldithiocarbazate to afford the corresponding 6,8-dimethyl chromeno[4,3-*c*]pyrazol-4(2*H*)-one (**24**) (Scheme 7) [35,36].

**Scheme 8:**
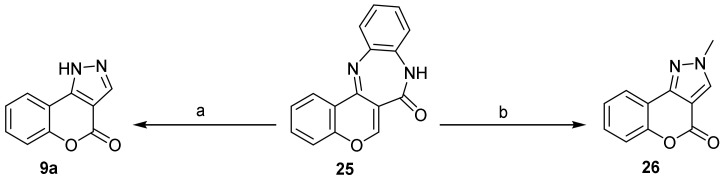
[1]Benzopyrano[4,3-c][1,5]benzodiazepin-7(8*H*)-one in the synthesis of chromeno[4,3-*c*]pyrazol-4(2*H*)-ones **9a** and **26**. Reagents and conditions. (**a**) NH_2_NH_2_.H_2_O, AcOH, reflux, 10 min, 48% yield; (**b**) NH_2_NHCH_3_, AcOH, reflux, 10 min, 48% yield.

Another synthetic pathway was performed by the dissociation of a large molecule in an acidic medium. [1]Benzopyrano[4,3-c][1,5]benzodiazepin-7(8*H*)-one (**25**) was prone to lose *o*-phenylenediamine in a reaction with some *N*-binucleophiles, such as hydrazine and methyl hydrazine that led exclusively to chromeno[4,3-*c*]pyrazol-4(2*H*)-ones **9a** and **26** (Scheme 8) [37].

Pyrone Construction

**Scheme 9:**
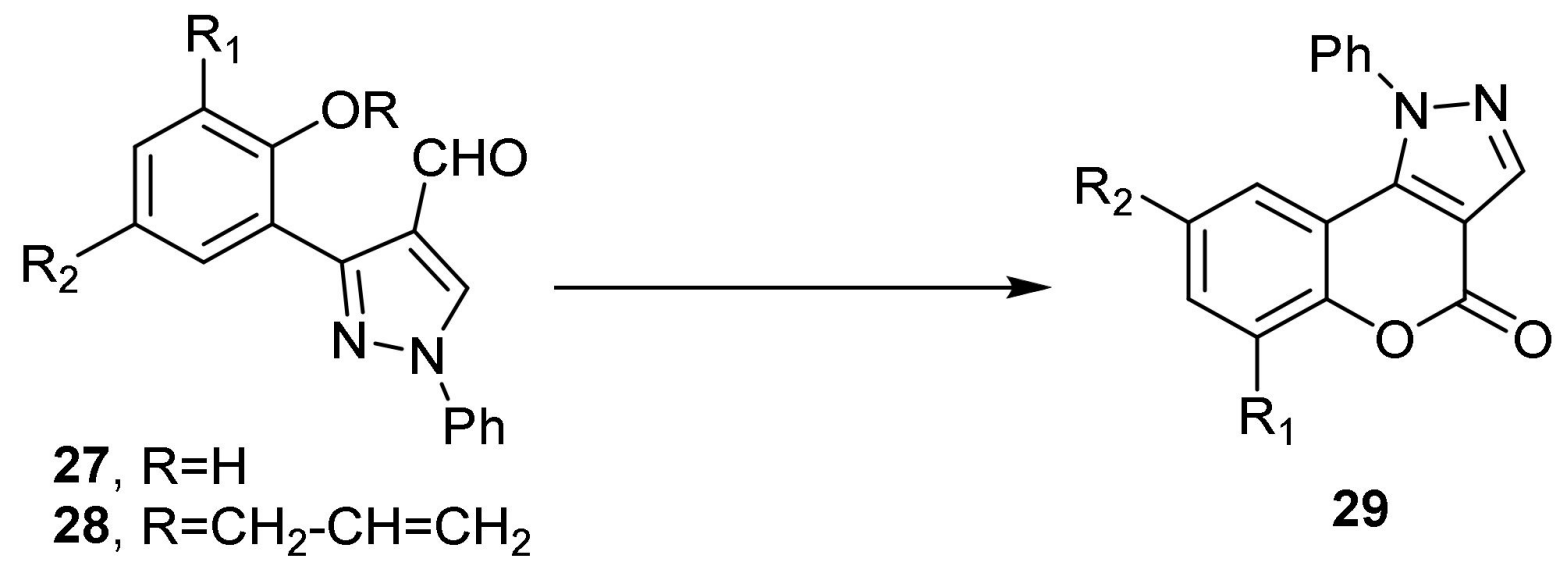
Synthesis of 2-phenyl-chromeno[4,3-*c*]pyrazol-4(1*H*)ones **29**. Reagents and conditions. R = H, or CH_2_-CH=CH_2_, 10 mol%, DMSO, conc. H_2_SO_4_, 120 °C, 5 h, seven outputs with 88–93% yield.

Lokhande et al. introduced a simple and convenient method for the synthesis of fused chromeno[4,3-*c*]pyrazol-4(2*H*)-one. It was accomplished using iodine catalyzed oxidative cyclization of 3-(2-hydroxyaryl)-1-phenyl-1*H*-pyrazole-4-carbaldehydes **27** or 3-(2-(allyloxyaryl)-1-phenyl-1*H*-pyrazole -4-carbaldehydes **28** in dimethyl-sulfoxide that supplied the corresponding 2-phenyl-pyrazolo[4,3-*c*]coumarins **29** (Scheme 9) [38]. The reaction was initially performed with (5%) of iodin in dimethyl-sulfoxide in the presence of H_2_SO_4_ at 60 °C using known methods, but the reaction did not take place. To overcome this problem, the reaction was carried out by increasing the iodine ratio to 10% and raising the temperature to 120 °C, which gave good results. This pathway has an advantage over the previous pyrazole construction methods which were relatively unstable and gave a mixture of isomeric 1-aryl and 2-arylpyrazolo[4,3-*c*]coumarins [25,39].

**Scheme 10:**
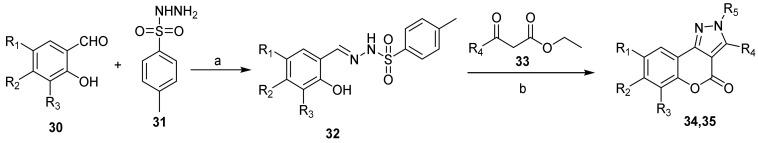
Synthesis of chromeno[4,3-*c*]pyrazol-4(1*H*)ones **34** and **35**. Reagents and conditions. (**a**) CH_3_CN, 2 h, r.t.; (**b**) La(OTf)_3_, 130 °C, 8 h; 25 outputs with 22–78% yield.

Raju and his co-workers described the synthetic strategy for chromeno[4,3-*c*]pyrazol-4(1*H*)ones **34** and 2-tosyl-chromeno[4,3-*c*]pyrazol-4(1*H*)ones **35.** The pathway started from a reaction between salicylaldehydes **30** and *p*-toluenesulfonyl hydrazide **31.** It proceeded towards salicylaldehyde tosylhydrazone **32** which was in situ reacted with 3-oxobutanoates **33** in presence of lanthanum tris(trifluoromethanesulfonate) to deliver the desired coumarin (Scheme 10) [40].

Chromeno[3,4-c]pyrazol-4(2*H*)-one; (1*H*-Benzopyrano[3,4-c]pyrazole)
Pyrazole Construction

**Scheme 11:**
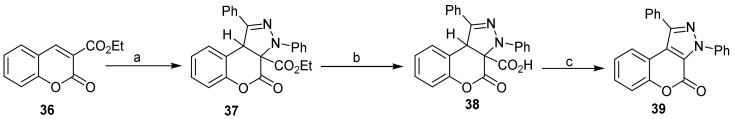
Synthesis of 1,3-diphenyl-chromeno[3,4-*c*]pyrazol-4(1*H*)one (**39**). Reagents and conditions. (**a**) Diphenylnitrilimine (DPNI), benzene, TEA, 65% yield; (**b**) KOH (10%), 1 h, reflux, 80% yield; (**c)** heat, 98% yield.

The literature reported numerous synthetic routes for chromeno[3,4-*c*]pyrazol-4(2*H*)-one. A simple pathway beginning from the 1,3-dipolar cycloaddition of diphenylnitrilimine (DPNI) to 3-ethoxy carbonyl coumarin (**36**) to build the pyrazole moiety **37**. Treatment of the ester (**37)** with an aqueous solution of potassium hydroxide (10%) gave the corresponding acid (**38**). Decarboxylation of **37** was accompanied by dehydrogenation which facilitated the attainment of 1,3-diphenyl-chromeno[3,4-*c*]pyrazol-4(1*H*)one (**39**) in a considerable yield (Scheme 11) [41].

**Scheme 12:**
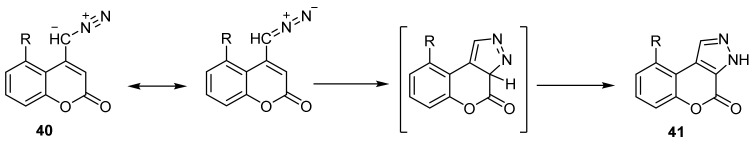
Thermal conversion of 4-diazo-methyl coumarin. Reagents and conditions. Toluene, heat, stirring, R = H: 85% yield, R = Me: 98% yield.

On the other hand, thermal conversion of 4-diazo-methyl coumarins **40** into the corresponding chromeno[3,4-*c*]pyrazol-4(2*H*)-ones **41** in toluene followed the pathway depicted in Scheme 12 [42,43]. The presence of the alkyl substituent at the peri position to the attached diazo-methyl group in coumarins markedly destabilized the diazo-methyl group and facilitated the pyrazole isomerization.

**Scheme 13:**
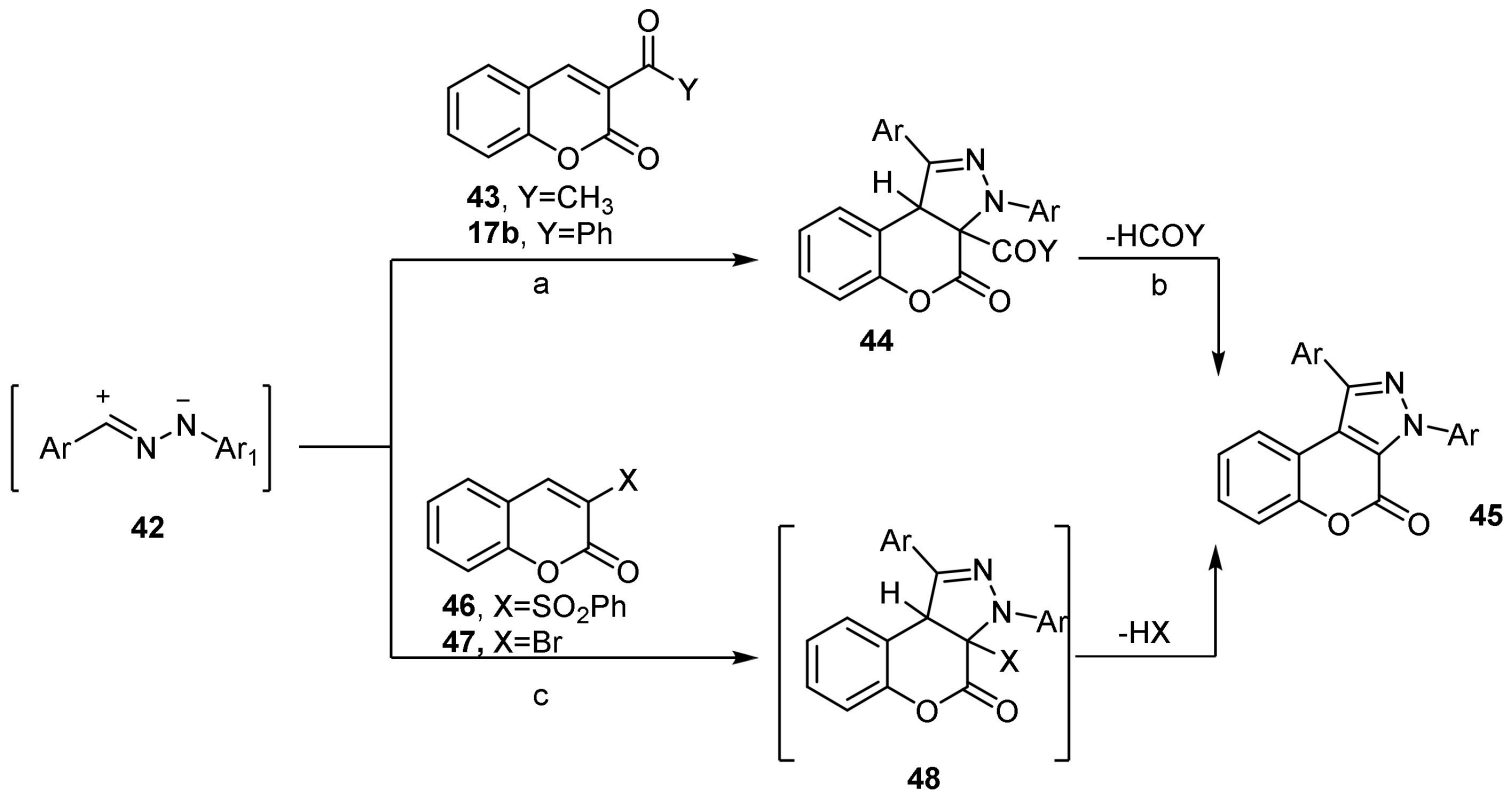
The role of hydrazonyl halides in the preparation of chromeno[3,4-*c*]pyrazol-4(2*H*)-ones. Reagents and conditions. (**a**) Benzene, reflux, TEA, two outputs Y = CH_3_ (51%), Y = Ph (48%); (**b**) i: KOH (10%), 12 h, reflux; ii: toluene, 2 h, reflux; (**c**) benzene, reflux, TEA, two outputs Ar = C_6_H_4_F-*p*, Ar_1_ = C_6_H_4_NO_2_-*p* (48%), Ar = C_6_H_3_Cl_2_-*2,4*, Ar_1_ = C_6_H_4_NO_2_-*p* (51%).

Hydrazonyl halides played an important role in the preparation of chromeno[3,4-*c*]pyrazol-4(2*H*)-ones [11]. This coumarin was accessible from the reaction of hydrazonyl halides **42** with 3-acetylcoumarin (**43**) or 3-benzoylcoumarin (**17b**) in the presence of triethylamine. Subsequently, the dihydro-products **44** were refluxed in aqueous potassium hydroxide (10%) solution, and toluene successively to give chromeno[3,4-*c*]pyrazol-4(2*H*)-ones **45** (Scheme 13). Alternatively, compounds **45** were synthesized via cycloaddition of the hydrazonyl halides **42** to the 3-phenylsuphonylcoumarin (**46)**, or the 3-bromocoumarin (**47).** The thermal elimination of benzenesulfinic acid or hydrogen bromide from the corresponding cyclo-adducts **48** resulted in the target **45** (Scheme 13).

##### Imidazole

**Figure 2:**
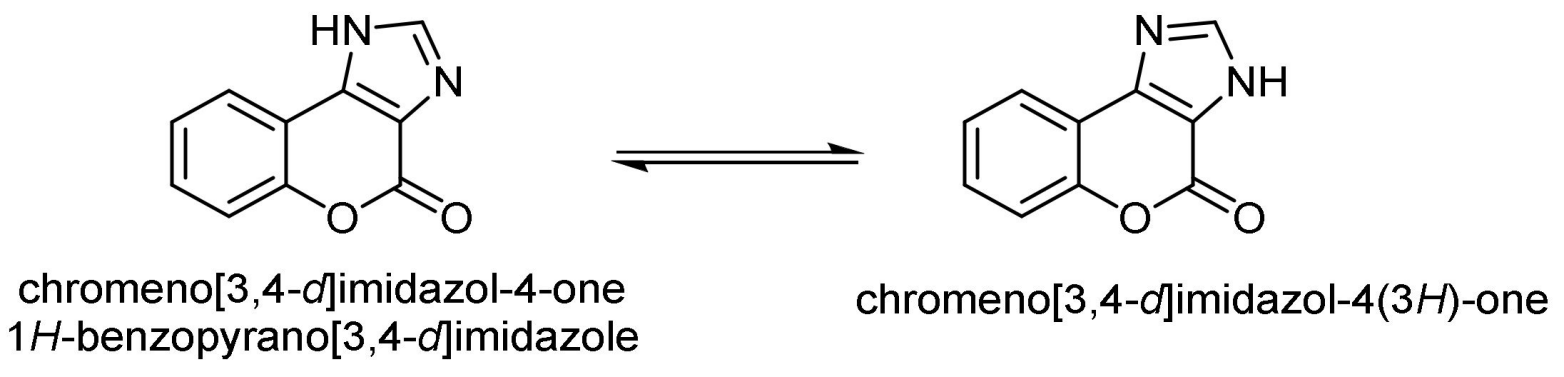
The common isomer of fused chromeno-imidazole.

Fusion of the imidazole ring with the pyrone ring of coumarin leads to one structural isomer, namely chromeno[3,4-*d*]imidazol-4-one, chromeno[3,4-*d*]imidazole-4(3*H*)-one, (1*H*-benzopyrano[3,4-*d*]imidazole) (Figure 2).

Chromeno[3,4-d]imidazol-4-one; (1*H*-Benzopyrano[3,4-d]imidazole)
Imidazole Construction

**Scheme 14:**
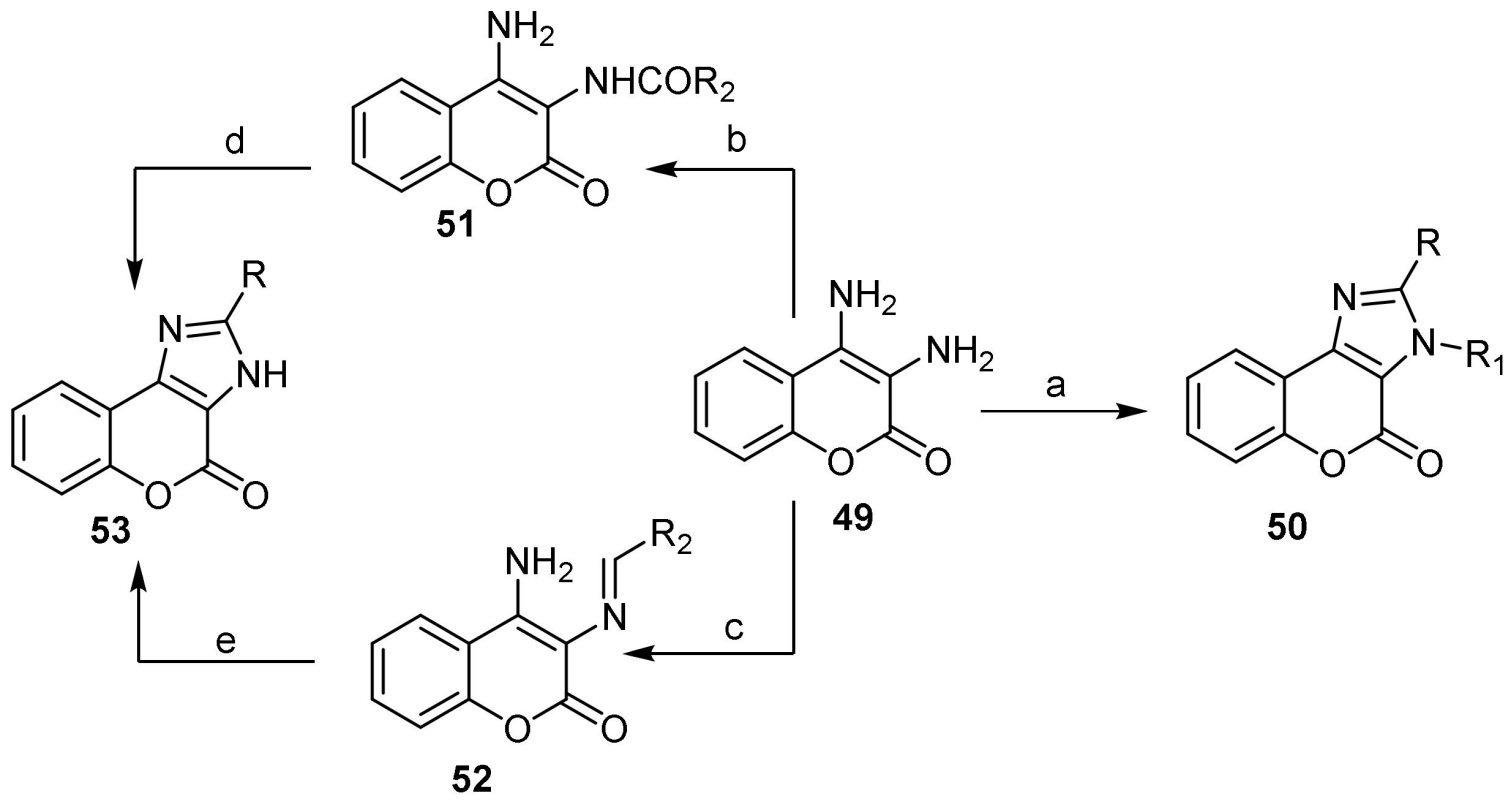
Simple synthetic routes of novel 1*H*-benzopyrano[3,4-*d*]imidazoles **50** and **53**. Reagents and conditions. (**a**) i: Formic acid, conc. HCl, heating, 12 h, R = R_1_ = H, 87% yield; ii: glacial AcOH, conc. HCl, heating, 12 h, R = CH_3_, R_1_ = H, 92% yield; iii: HCHO, EtOH, conc. HCl, heating, 2 h, R = H, R_1_ = CH_3_, 82% yield; (**b**) (AcO_2_)_2_O or RCOCl, benzene, heat, 40 °C, 1 h, 4 outputs with 50–84% yield; (**c**) appropriate aldehyde, EtOH, reflux, 1 h, 4 outputs with 59–81% yield; (**d**) oil bath, 310–320 °C, 30 min; (**e**) lead tetraacetate, benzene, stirring, r.t., 2 h, 4 outputs with 59–91% yields.

Few synthetic routes to prepare benzo-pyrano-imidazoles were reported. The main synthetic approach involved the condensation of 3,4-diaminocoumarin (**49**) with different reagents [7,44]. Kitan and his coworkers elaborated a simple synthetic route of novel 1*H*-benzopyrano[3,4-*d*]imidazole-4-one starting from the in situ prepared 3,4-diaminocoumarin (**49**) by catalytic hydrogenation of 4-amino-3-nitrocoumarin. The condensation of 3,4-diaminocoumarin (**49**) with different acids including formic acid, acetic acid, or formaldehyde in presence of concentrated hydrochloric acid established 1*H*-benzopyrano[3,4-*d*]imidazoles **50** (Scheme 14) [7]. On the other hand, different series of 3-*N*-(4-aminocoumarin-3-yl)aroylamides **51** and 3-*N*-arylidenamino-4-aminocoumarins **52** were prepared from 3,4-diaminocoumarin **49**. Cyclization of **51** and **52** under heating, or in the presence of lead tetraacetate resulted in the fused 2-substituted 4*H*-chromeno[3,4-*d*]oxazol-4-ones **53** (Scheme 14) [44].

**Scheme 15:**
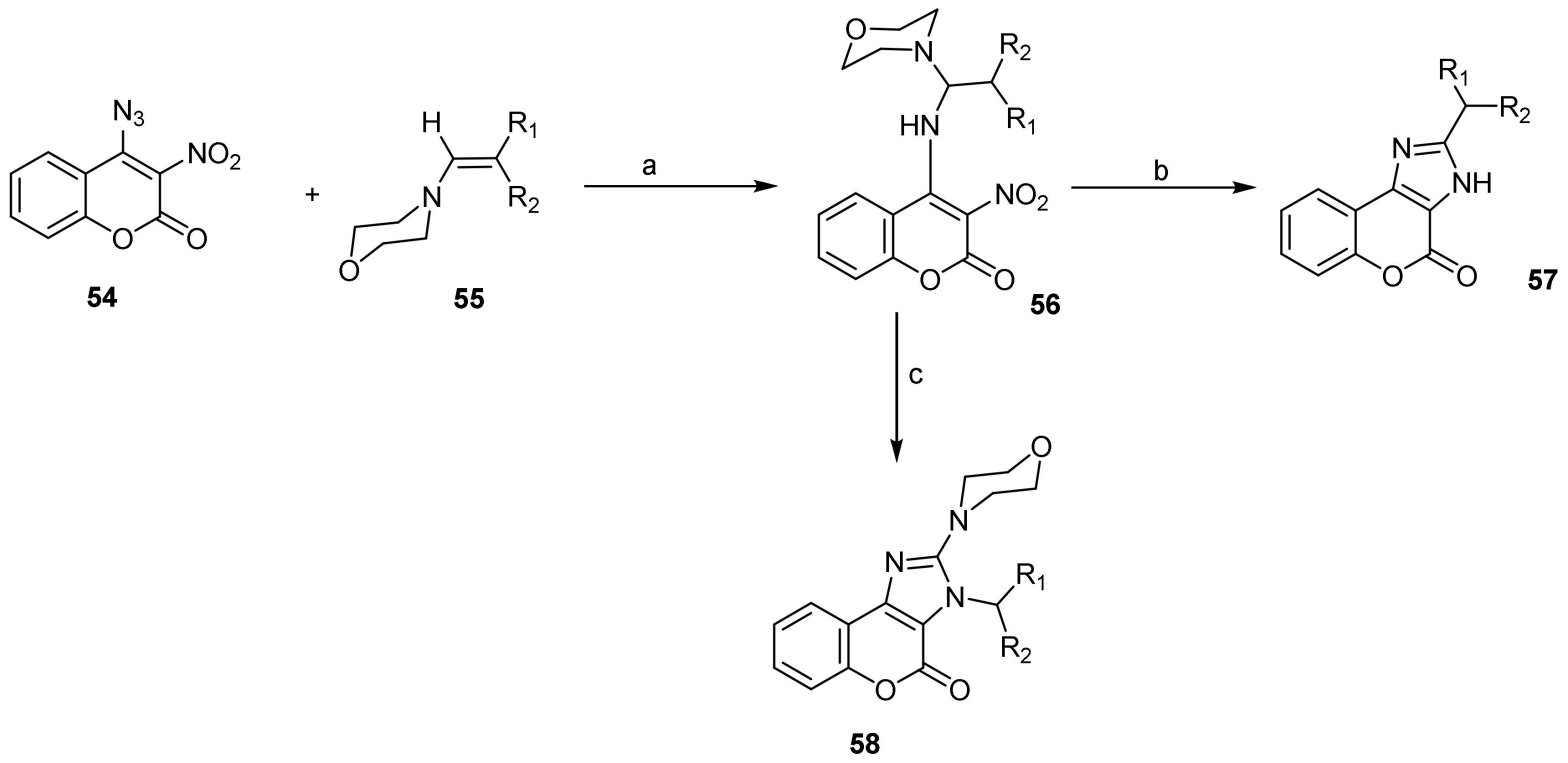
[1]Benzopyrano[3,4-d]imidazol-4(3H)-ones **57** and **58** bearing hydrogen or alkyl groups on N-3, and alkyl or amino substituents on C-2. Reagents and conditions. i, R_1_ = Ph, R_2_ = H; ii, R_1_ = 4-BrC_6_H_4_, R_2_ = H; iii, R_1_ = CH_2_Ph, R_2_ = H; iv, R_1_ = CH_2_CH_3_, R_2_ = H; v, R_1_ = R_2_ = CH_3_; (**a**) CH_2_Cl_2_, −40°C, five outputs with 67–89% yields; (**b**) NaBH_4_, Pd/C, MeOH, H_2_O, five outputs with 55–65% yield; (**c**) N_2_, P(OC_2_H_3_)_3_, reflux, five outputs with 57–65% yields.

Trimarco’s research group developed a new method to synthesize substituted [1]benzopyrano[3,4-*d*]imidazol-4(3*H*)-ones bearing hydrogen or alkyl groups on N-3, and alkyl or amino substituents on C-2 in good yields [45]. 2-Alkyl-[1]benzopyrano[3,4-*d*]imidazol-4(3*H*)-ones **57** were obtained from acetamidines **56** carrying a 3-nitrocoumarin group at N-1 by reduction with sodium borohydride/palladium 10% (Scheme 15) [45]. Benzopyranoimidazoles **58** of an amino substituent on C-2 were obtained by refluxing **56** in excess of triethyl phosphite (Scheme 15) [45].

#### 2.1.2. Two Different Heteroatoms

##### Thiazole and Isothiazole

**Figure 3:**
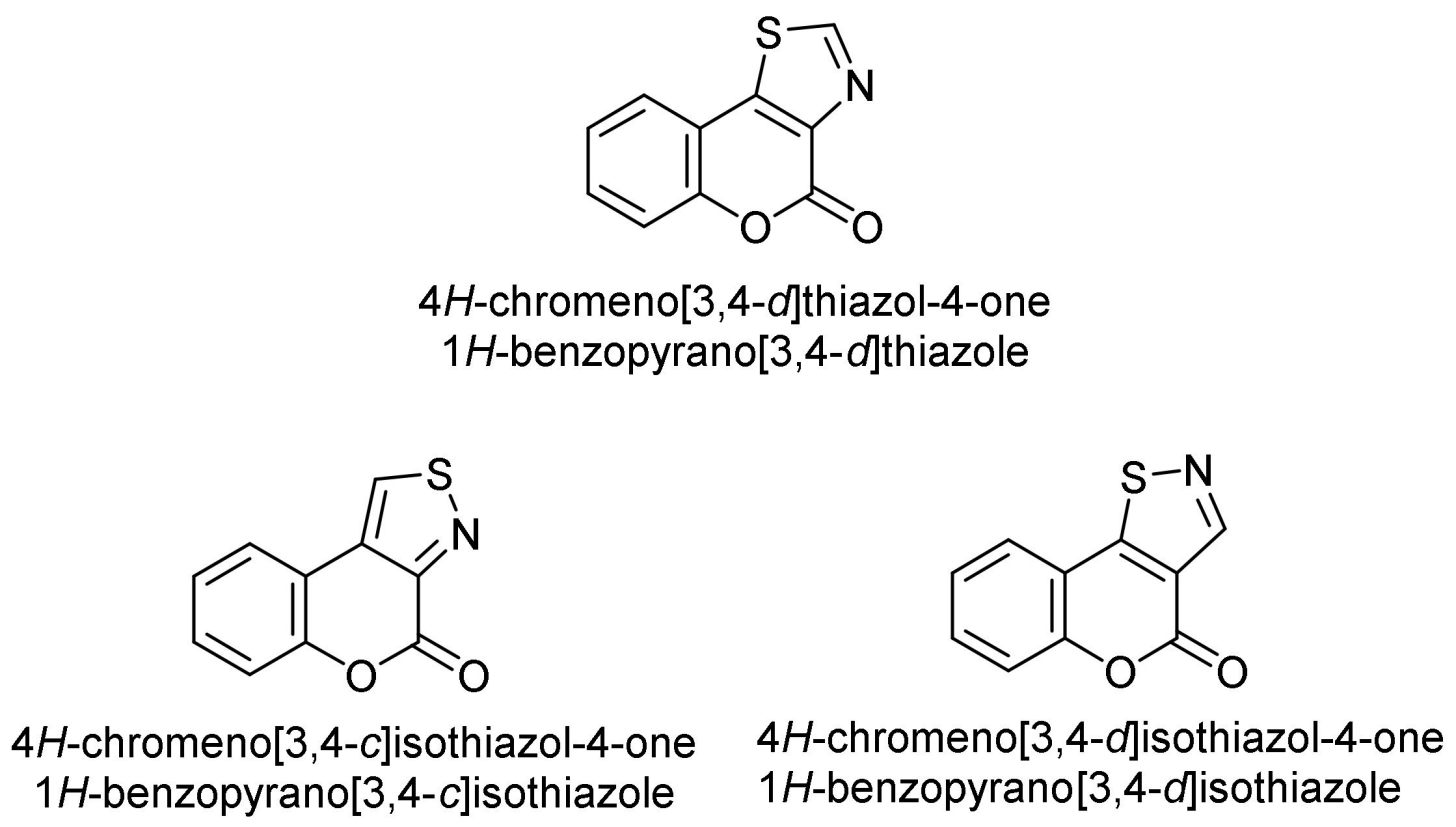
The common three isomers of fused the thiazole and isothiazole ring to the α-pyrone moiety of the coumarin.

4*H*-chromeno[3,4-*d*]thiazol-4-one (1*H*-benzopyrano[3,4-*d*]thiazole), 4*H*-chromeno[3,4-*c*]isothiazol-4-one (1*H*-benzopyrano[3,4-*c*]isothiazole), and 4*H*-chromeno[3,4-*d*]isothiazol-4-one (1*H*-benzopyrano[3,4-*d*]isothiazole) are isomers of the fused five-member ring (containing N and S atoms) with the pyrone ring of coumarin (Figure 3).

4*H*-Chromeno[3,4-d]thiazol-4-one
Thiazole Construction

**Scheme 16:**
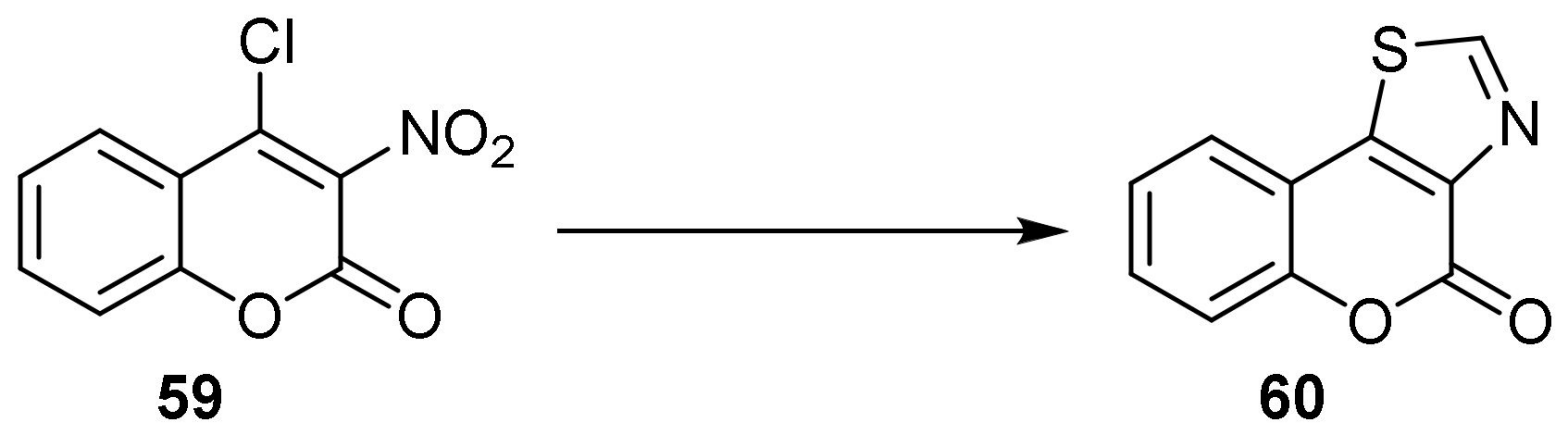
Synthesis of 4*H*-chromeno[3,4-*d*]thiazol-4-one (**60**). Reagents and conditions. EtOH, NaHSO_3_, pH 3, reflux, 3 h, 78% yield.

The reaction of 4-chloro-3-nitrocoumarin (**59**) with carbon disulfide in ethanol in the presence of sodium hydrogen sulfate produced 4*H*-chromeno[3,4-*d*]thiazol-4-one (**60**) (Scheme 16) [7].

**Scheme 17:**
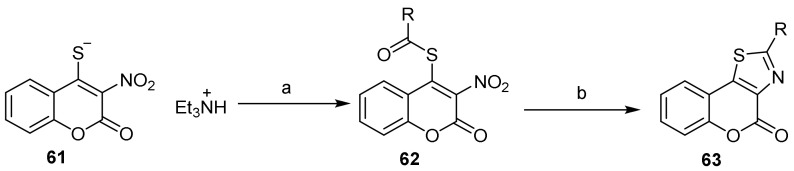
Synthetic pathway of 2-aryl-4*H*-chromeno[3,4-*d*]thiazol-4-ones **63**. Reagents and conditions. (**a**) THF, stirring, 30 min, five outputs with 40–73% yield; (**b**) Fe, conc. AcOH, reflux, 60 °C for 1 h, 90 °C for 3–4 h, five outputs with 45–95% yield.

In situ prepared 4-aroylthio-3-nitrocoumarins **62** which was obtained from the reaction of 4-mercapto-3-nitrocoumarin (**61**) with different aroyl chlorides were allowed to cyclize in the presence of iron and glacial acetic acid that gave 2-aryl-4*H*-benzopyrano[3,4-*d*]thiazol-4-ones **63** (Scheme 17) [44].

**Scheme 18:**
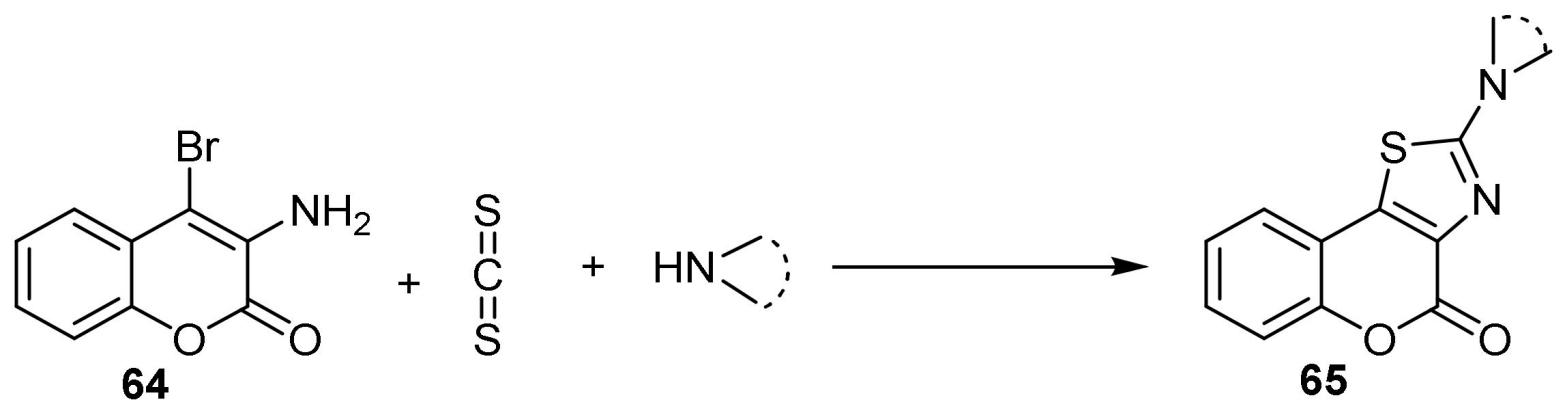
Synthetic pathway of 2-amino-4*H*-chromeno[3,4-*d*]thiazol-4-ones **65**. Reagents and conditions. CS_2_ (1.2 mmol) and K_2_CO_3_ (3.0 mmol), FeCl_3_ (1.5 mmol), 110 °C, 6–8 h, N_2_, pyrrolidine 64% yield, *N*-ethylaniline 74% yield.

Anwar et al. employed a facile, green, and efficient, one-pot multicomponent reaction (MCR) catalyzed by iron(III) chloride to synthesize the coumarin annulated 2-aminothiazole (**65)** (Scheme 18) [46].

**Scheme 19:**
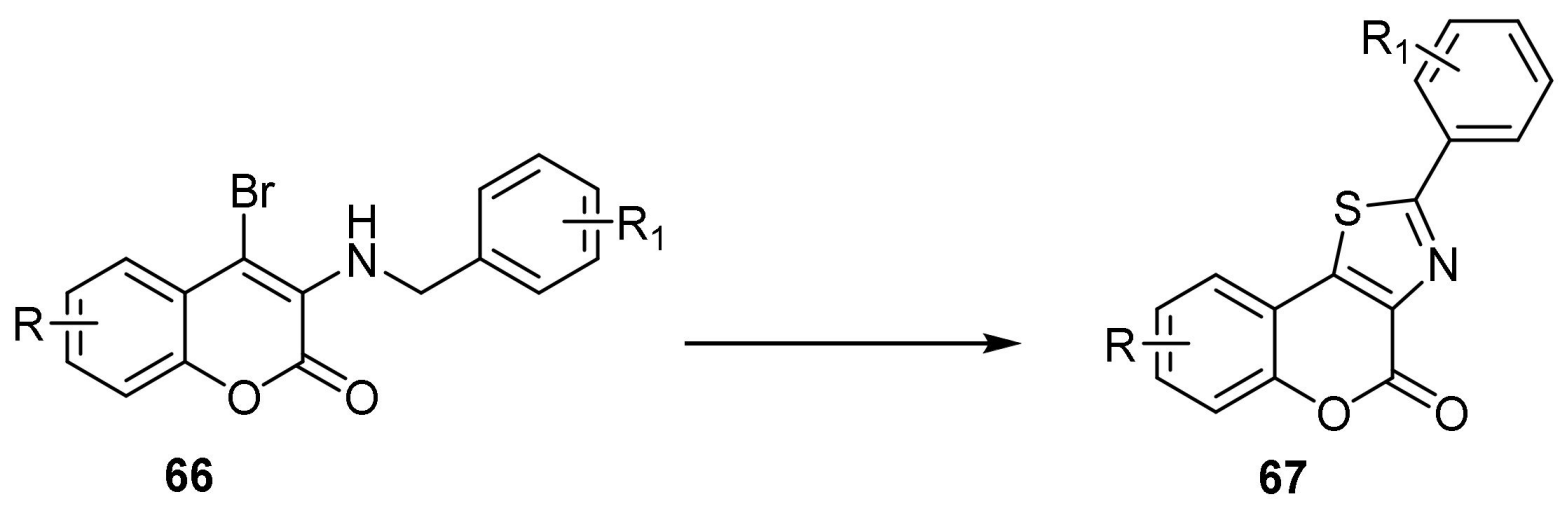
Oxidative cross-coupling reaction to synthesis of 4*H*-chromeno[3,4-*d*]thiazol-4-ones **67**. Reagents and conditions. Na_2_S (3 equiv.), I_2_ (20 mmol%), 30% aq. H_2_O_2_ (5 equiv), DMF, 120 °C, 22 outputs with 65–89% yields.

Transition metal-free oxidative coupling for the formation of C–S bonds was employed to synthesize different 4*H*-chromeno[3,4-*d*]thiazol-4-ones. The key point in C–H bond activation of 3-(benzylamino)-4-bromo-substituted chromenone derivatives **66** was the presence of sodium sulfide as a source of sulfur, and iodine as a catalyst. The terminal oxidation was performed by H_2_O_2_ to form various 2-phenyl-4*H*-chromeno(3,4-*d*)thiazol-4-one derivatives **67** (Scheme 19) [47].

4*H*-Chromeno[3,4-c]isothiazol-4-one
Pyrone Construction

**Scheme 20:**
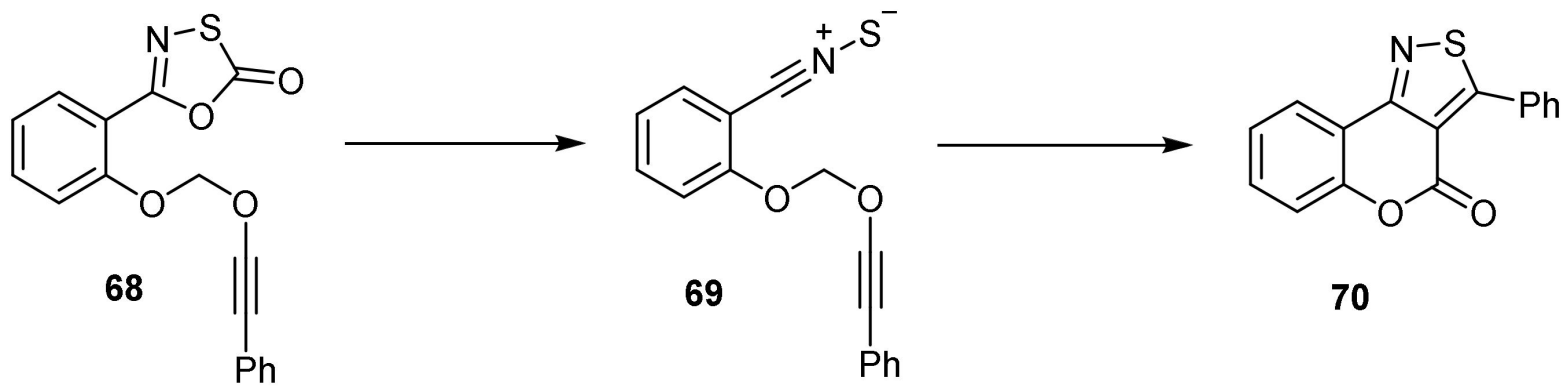
Synthesis of 4-oxo-3-phenyl-4*H*-chromeno[4,3-*c*]isothiazole (**70**). Reagents and conditions. Xylene, heat, 16 h, 70% yield.

The 1,3-dipolar cycloaddition reactions of nitrile sulfides (RC-N≡S-) played a particular role in the synthesis of 4*H*-chromeno[4,3-*c*]isothiazole [48,49,50]. For example, heating of acetylenic oxathiazolone (**68**) in xylene afforded 4-oxo-3-phenyl-4*H*-chromeno[4,3-*c*]isothiazole (**69**) [48]. The initial step of the reaction was decarboxylation of the oxathiazolone followed by intramolecular 1,3-dipolar cycloaddition of the resulting nitrile sulfide (**70**) to the adjacent alkyne (Scheme 20).

**Scheme 21:**
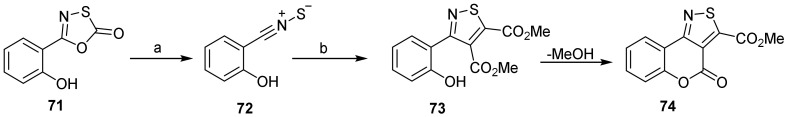
Synthesis of methyl 4-oxo-4*H*-chromeno[4,3-*c*]isothiazole-3-carboxylate (**74**). Reagents and conditions. (**a**) Heat, -CO_2_, 84% yield; (**b**) EtOAc, DMAD,10 min, 160 °C, microwave, 94% yield.

In 2010, Fordyce et al. improved a synthetic approach of 4*H*-chromeno[4,3-*c*]isothiazole as a result of the 1, 3-dipolar cycloaddition reactions of *o*-hydroxybenzonitrile sulfide (**72**), generated by microwave-assisted decarboxylation of oxathiazolone (**71**) [51]. The reaction of the *o*-hydroxyphenyloxathiazolone (**73**) with dimethyl acetylenedicarboxylate DMAD (1:2) in ethyl acetate supplied methyl 4-oxo-4*H*-chromeno[4,3-*c*]isothiazole-3-carboxylate (**74**) (Scheme 21).

**Scheme 22:**
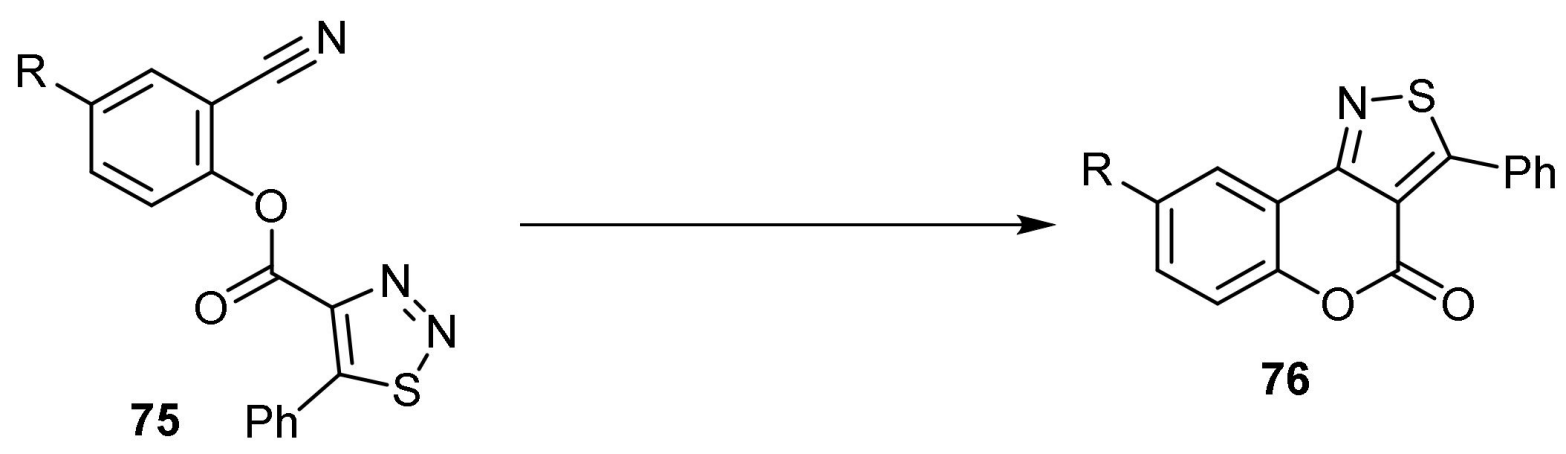
Synthesis of 8-substituted-3-phenyl-4*H*-chromeno[4,3-*c*]isothiazol-4-ones **76**. Reagents and conditions. An amount of 5.0 mol% [Rh(COD)Cl]_2_, 12.0 mol% DPPF, PhCl (1.0 mL), N_2_, 80 °C; R = H 90% yield; R = Me 99% yield; R = Br 90% yield.

In 2017, Lee and his coworkers reported an efficient intramolecular Rh-catalyzed transannulation of thiadiazoles linked to cyanoalkoxycarbonyl. The ring closure of compound 75 was catalyzed by 1,1′-bis(diphenylphosphino) ferrocene DPPF to form the corresponding 8-substituted-3-phenyl-4*H*-chromeno[4,3-*c*]isothiazol-4-ones **76** in good yields of 90–99% (Scheme 22) [52].

4*H*-Chromeno[3,4-d]isothiazol-4-one

**Scheme 23:**
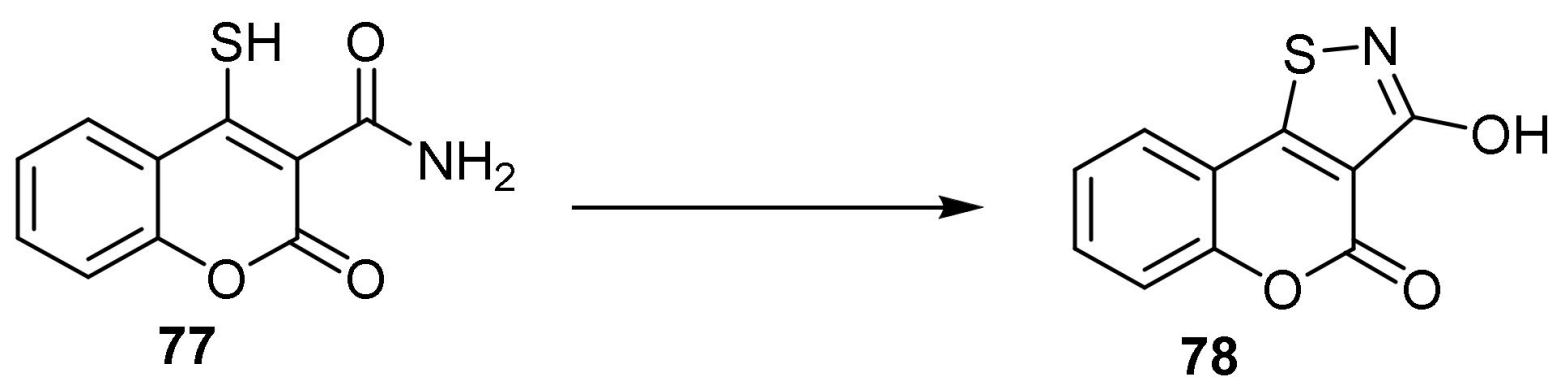
Synthesis of 4*H*-chromeno[3,4-*d*]isothiazol-4-one (**78**). Reagents and conditions. Br_2_, EtOAc, heat, 4 h, 96% yield.

Up to our knowledge, only one article discussed the synthesis of 4*H*-chromeno[3,4-*d*]isothiazol-4-one [53]. The reported method included cyclization of 4-mercapto-2-oxo-2*H*-chromene-3-carboxamide (**77**) on heating with bromine in ethyl acetate to form 2-hydroxy-4*H*-chromeno[3,4-*d*]isothiazol-4-one (**78**) (Scheme 23).

##### Oxazole and Isoxazole

**Figure 4:**
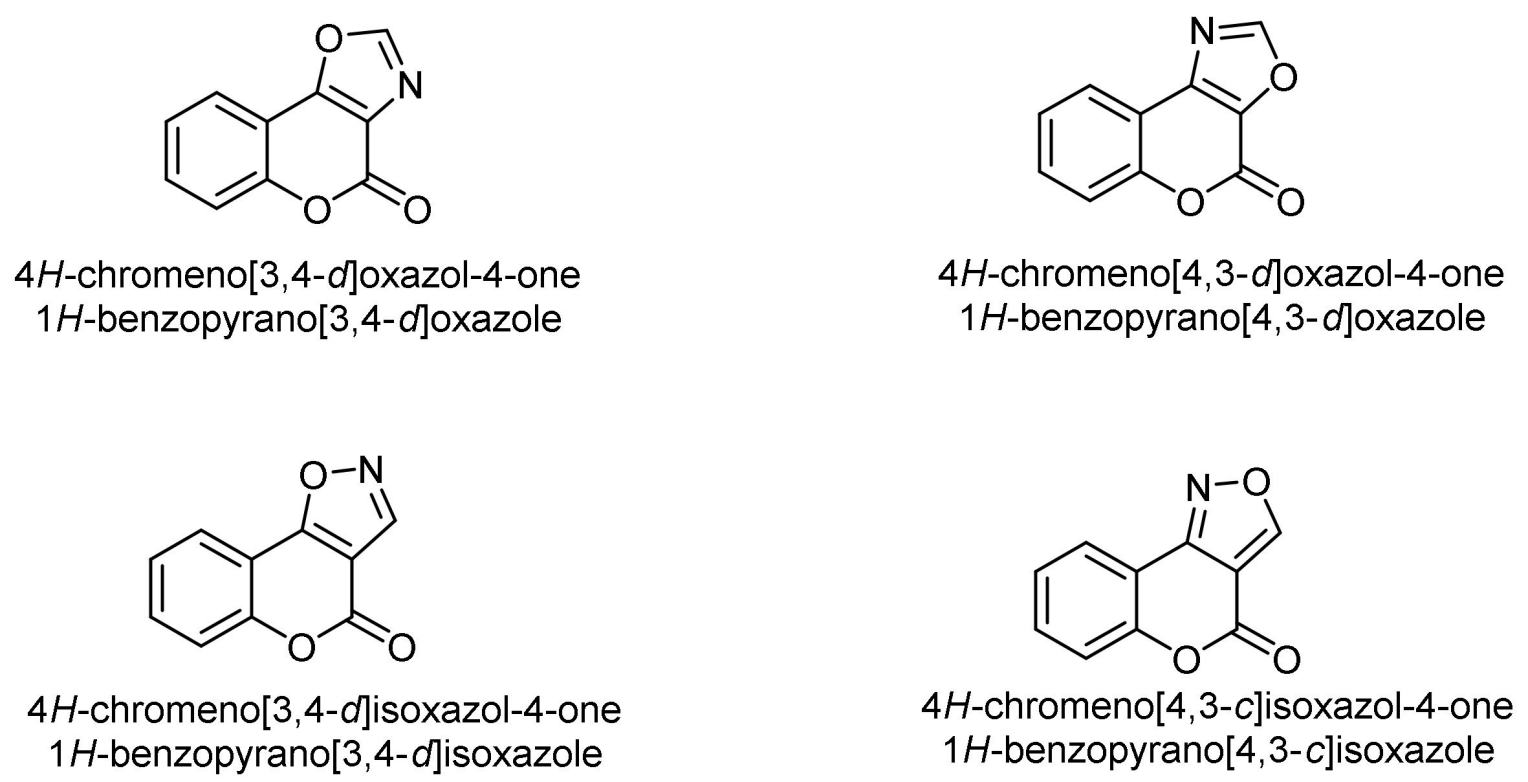
The common four isomers of fused oxazole and isoxazole ring to the α-pyrone moiety of the coumarin.

Fusion of a five-member ring containing (N and O atoms) with the pyrone ring of coumarin leads to four structural isomers, viz. 4*H*-chromeno[3,4-*d*]oxazol-4-one, 4*H*-chromeno[4,3-*d*]oxazol-4-one, 4*H*-chromeno[3,4-*d*]isoxazol-4-one and 4*H*-chromeno[4,3-*c*]isoxazol-4-one (Figure 4).

4*H*-Chromeno[3,4-d]oxazol-4-one
Oxazole Construction

**Scheme 24:**
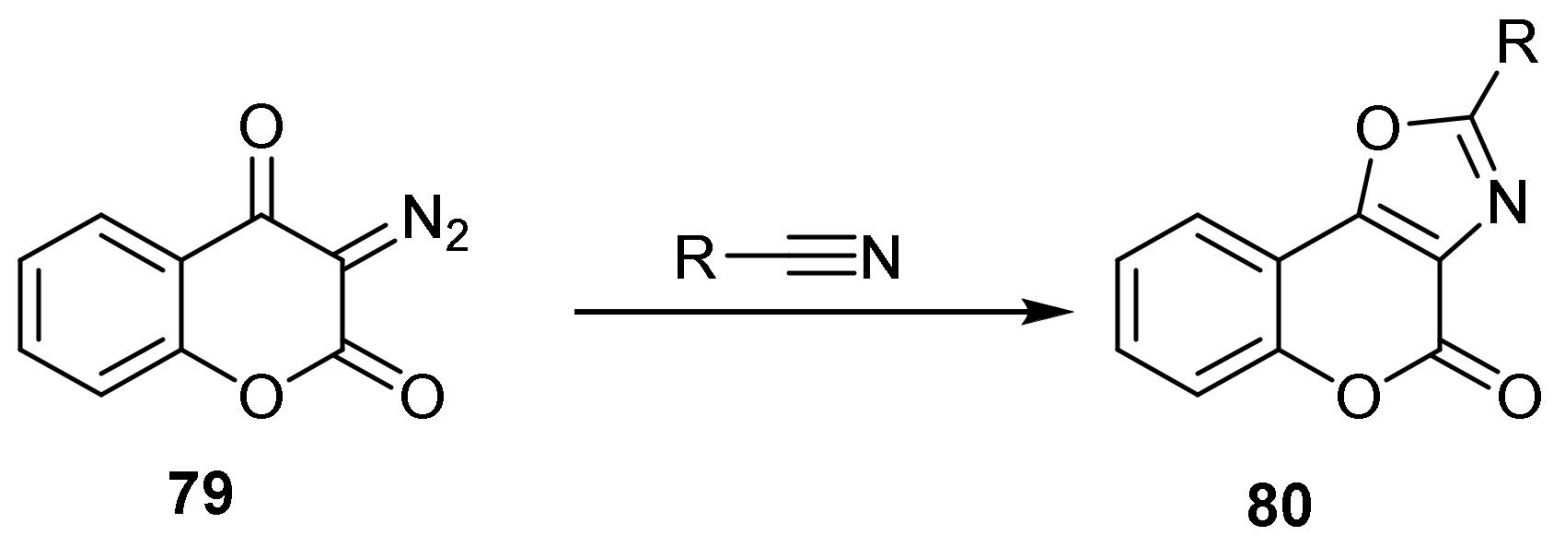
3-Diazo-2,4-chromenedione in the preparation of 4*H*-chromeno[3,4-*d*]oxazol-4-ones **72**. Reagents and conditions. Rh_2_(OAc)_4_, 60 °C, 5 h, three outputs, R = CH_3_ 50% yield, R = CH_2_Cl 95% yield, R = CH_2_Ph 73% yield.

Rhodium(II)-catalyzed reactions of 3-diazo-2,4-chromenedione (**79**) with several nitriles, such as acetonitrile, chloroacetonitrile and phenylacetonitrile established 2-substituted-4*H*-chromeno[3,4-*d*]oxazol-4-ones **80** (Scheme 24) [54]. The 3-diazo-2,4-chromenedione (**79**) was prepared by the diazo-transfer reaction of the corresponding 4-hydroxycoumarin (**4**) with mesyl azide according to Taber’s method [55].

**Scheme 25:**
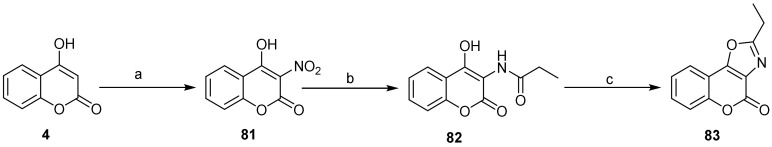
3-Nitrocoumarin in the preparation of 4*H*-chromeno[3,4-*d*]oxazol-4-one (**83**). Reagents and conditions. (**a**) HNO_2_, glacial AcOH; (**b**) Raney Ni, CH_3_CH_2_COOCOCH_2_CH_3_; (**c**) acetic acid anhydride, heat, 96% yield.

3-Nitro-4-hydroxycoumarin was crucial for the preparation of 2-substituted-4*H*-chromeno[3,4-*d*]oxazol-4-one. In 1961, Dallacker et al. reported the preparation of 3-nitrocoumarin (**73**) from 4-hydroxycoumarin (**4**) upon nitration with nitric acid in glacial acetic acid. Reduction of **81** by Raney nickel in presence of propionic anhydride afforded the corresponding *N*-(4-hydroxy-2-oxo-2*H*-chromen-3-yl)- propionamide (**82**). Intramolecular cyclization of amide **82** by heating in acetic anhydride afforded 2-ethyl-4*H*-chromeno[3,4-*d*]oxazol-4-one (**83**) (Scheme 25) [56].

**Scheme 26:**
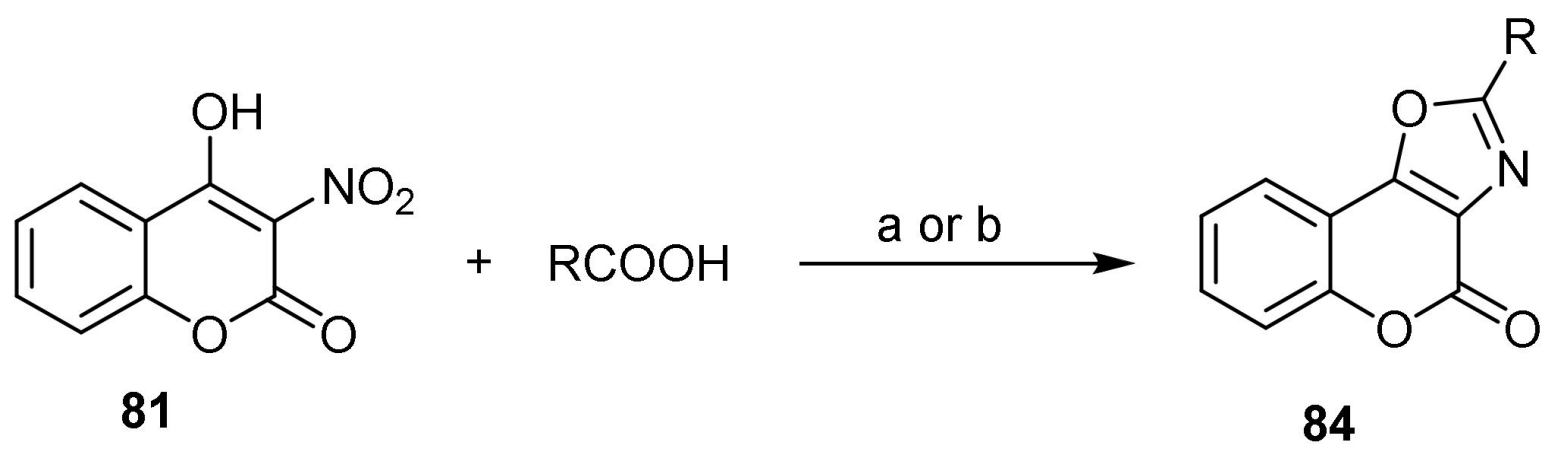
One-pot synthesis of 2-substituted 4*H*-chromeno[3,4-*d*]oxazol-4-ones **84**. Reagents and conditions: (**a**) PPh_3_ (2.5 equiv), P_2_O_5_ (4 equiv), MW irradiation, 130 °C or 140 °C, 1.5 h; (**b**) 5 mole % Pd/C (10%), H_2_ 1 atm, r.t., 1–3 h, then P_2_O_5_ (4 equiv), MW irradiation, 130 °C, 1 h, ten outputs with up to 91% yield.

In 2018, Litinas and his coworkers summarized the previous scheme in a green chemistry methodology [57]. They described the reaction of 4-hydroxy-3-nitrocoumarin (**81**) with acids in a one-pot reaction in the presence of PPh_3_ and P_2_O_5_ under microwave irradiation. Another one-pot two-step reaction was accomplished in the presence of Pd/C and hydrogen, followed by treatment with P_2_O_5_ under microwave irradiation (Scheme 26) [57].

Pyrone and Oxazole Construction

**Scheme 27:**
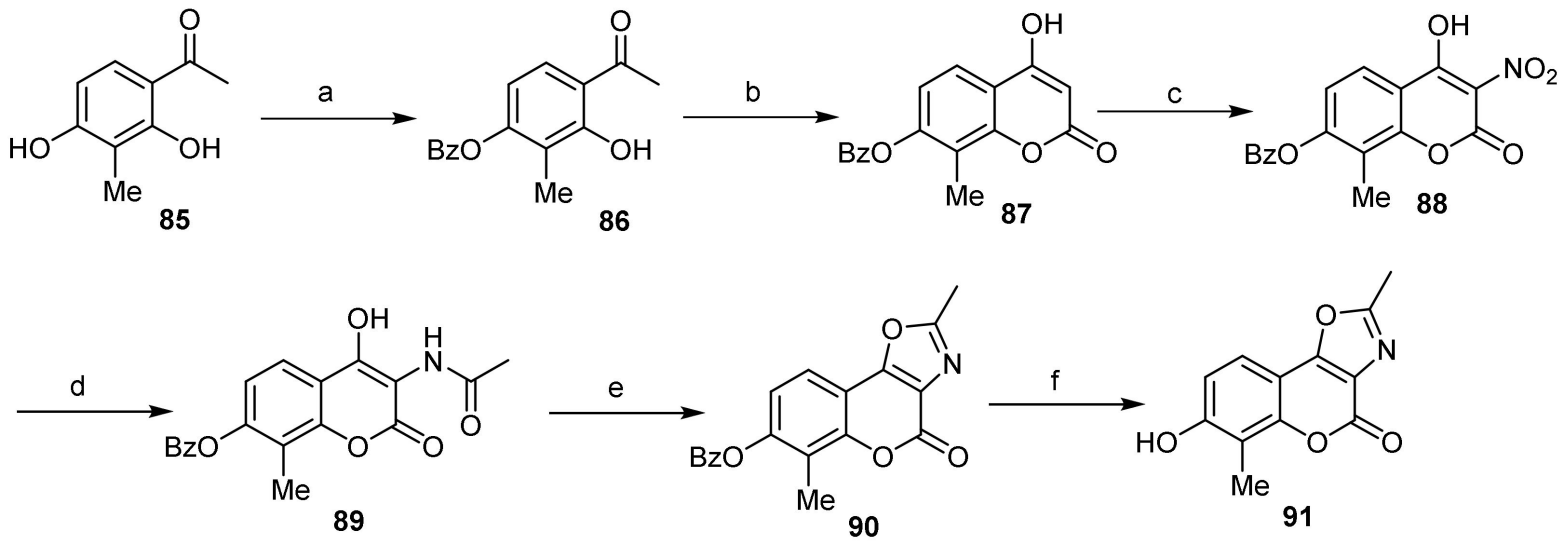
Synthesis of 7-hydroxy-2,6-dimethylchromeno[3,4-*d*]oxazol-4-one (**91**). Reagents and conditions: (**a**) BnCl, K_2_CO_3_, KI, acetone, 56 °C, 88% yield; (**b**) NaH, CO(OEt)_2_, toluene, 110 °C, 76%yield; (**c**) HNO_3_, H_2_SO_4_, CHCl_3_, room temperature, 93% yield; (**d**) Zn, AcOH, 110 °C, 86% yield; (**e**) POCl_3_, pyridine, THF, 66 °C, 87% yield; (**f**) 10% Pd/C, H_2_, THF/CH_2_Cl_2_, room temperature, 74% yield.

Wilson and his coworkers reported a high yield six-step synthesis of 7-hydroxy-2,6-dimethylchromeno[3,4-*d*]oxazol-4-one (**91**) from commercially available 2,4-dihydroxy-3-methyl-acetophenone [58]. The chemoselective benzylation of 2,4-dihydroxy3-methylacetophenone (**85**) gave the corresponding 4-benzyloxy derivative (**86**). Compound **86** was converted into the 4-hydroxycoumarin derivative (**87**) using diethyl carbonate and sodium hydride. Nitration of **87** with fuming nitric acid in chloroform at room temperature afforded the nitro derivative (**88**). Reduction of **88** using zinc in refluxing acetic acid afforded 3-acetamido-4,7-dihydroxycoumarin (**89**). Cyclization of **89** was achieved using pyridine-buffered POCl_3_ in THF under the Robinson–Gabriel mechanism. Finally, 7-hydroxy-2,6-dimethylchromeno[3,4-*d*]oxazol-4-one (**91)** was attainable through the debenzylation of oxazole **90** (Scheme 27) [58].

4*H*-Chromeno[4,3-d]oxazol-4-one
Oxazole Construction

**Scheme 28:**
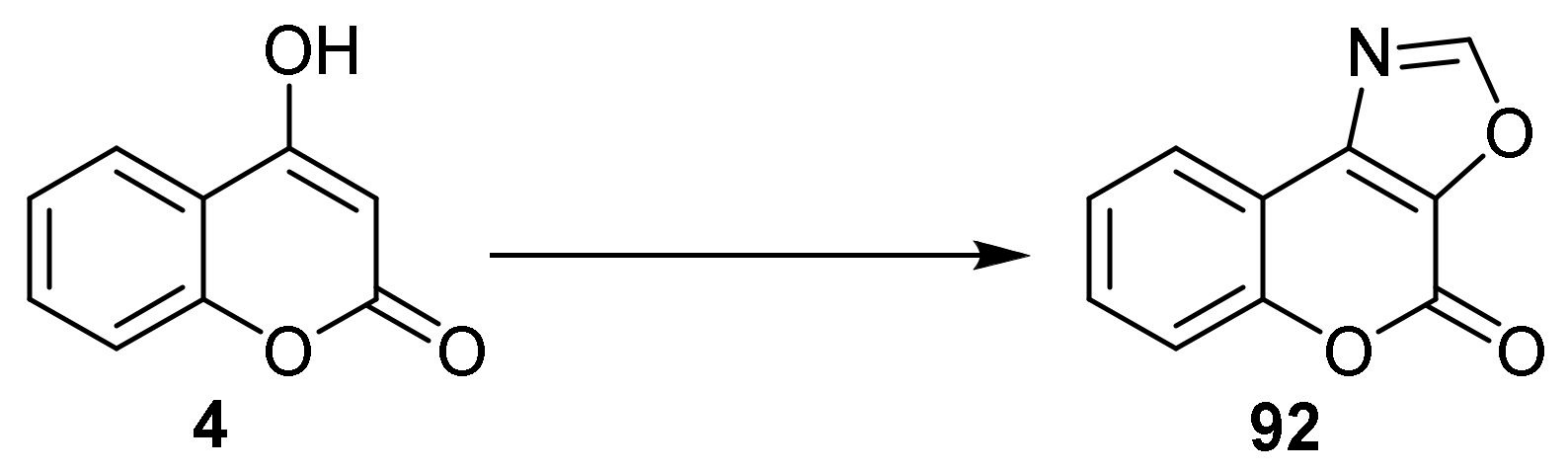
Synthesis of 4*H*-chromeno[4, 3-*d*]oxazol-4-one (**84**). Reagents and conditions. HCONH_2_, 160 °C, 84% yield.

Regarding 4*H*-chromeno[4,3-*d*]oxazole-4-one, only one article mentioned the preparation of such a fused system [59]. In 2004, Ray and Paul reported the synthesized 4*H*-chromeno[4,3-*d*]oxazole-4-one (**92**) via the reaction of 4-hydroxycoumarin (**4**) with formamide under reflux (Scheme 28).

4*H*-Chromeno[3,4-d]isoxazol-4-ones
Isoxazole Construction

**Scheme 29:**
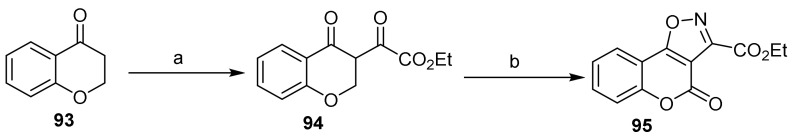
4-Chromanone in the synthesis of ethyl 4*H*-chromeno[3,4-*d*]isoxazol-3-carboxylate (**95**). Reagents and conditions. (**a**) Toluene, NaH, N_2_, stirring, r.t., 94% yield; (**b**) NH_2_OH.HCl, EtOH, reflux, 10 h, 91% yield.

4-Chromanone (**93**) was found to be one of the key compounds for the preparation of fused coumarin-isoxazole. 4-chromanone (**93**) was treated by a lower alkyl oxalate such as ethyl oxalate, in the presence of a suitable base (e.g., sodium amide, sodium methoxylate, or sodium hydride) in an anhydrous reaction medium. The obtained 4-oxo-chroman-3-glyoxylate (**94**) was refluxed with hydroxylamine hydrochloride in ethanol to create the fused isoxazole ring and ethyl 4*H*-chromeno[3,4-*d*]isoxazole-3-carboxylate (**95**) was formed (Scheme 29) [60].

**Scheme 30:**
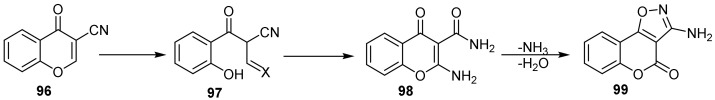
Synthesis of 2-amino-4*H*-chromeno[3,4-*d*]isoxazol-4-one (**99**). Reagents and conditions. NH_2_OH. HCl, NaOH, Ethanol, reflux, 45% yield.

Additionally, Sosnovskikh et al. studied the chemical transformation of the 3-cyanochromone (**96**) when reacted with hydroxylamine hydrochloride in basic condition. The cyclization at the CN group linked to the opened pyrone ring gave the corresponding 2-amino-3-carbamoylchromone (**98**). The re-cyclization of **98** exploiting another molecule of hydroxylamine led to the formation of 2-amino-4*H*-chromeno[3,4-*d*]isoxazol-4-one (**99**) (Scheme 30) [61].

**Scheme 31:**
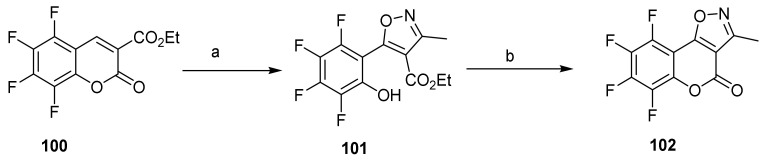
Synthesis of polyfluorochromeno[3,4-*d*]isoxazol-4-one (**102**). Reagents and conditions. (**a**) NH_2_OH. HCl, TEA, MeOH, 2 h, rt, 85% yield; (**b**) H_2_SO_4_, H_2_O, reflux, 1.5 h, 82% yield.

On the other hand, the reactivity of 3-ethoxycarbonyl-2-methyl substituted 5,6,7,8-tetrafluorochromone (**100**) toward hydroxylamine in an alkaline medium was explored [62]. Wherever the pyrone ring was firstly opened, hydroxylamine was involved in the isoxazole ring formation of **101**. Moreover, the cyclization to the corresponding 2-methyl-4H-6,7,8,9-tetrafluorochromeno[3,4-d]isoxazol-4-one (**102**) was performed by sulfuric acid treatment (Scheme 31) [62].

4*H*-Chromeno[4,3-c]isoxazol-4-one
Isoxazole Construction

**Scheme 32:**
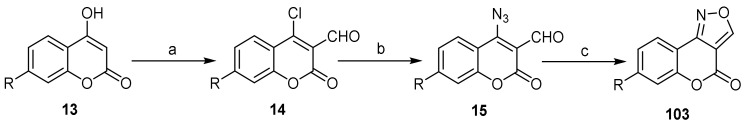
Synthetic pathway of 4*H*-chromeno[4,3-*c*]isoxazol-4-ones **103**. Reagents and conditions. (**a**) POCl_3_, DMF, H_2_O, four outputs with 65–80% yields; (**b**) NaN_3_, DMF, four outputs with 50–80% yield; (**c**) DMF, heat, 60–90 °C, -N_2_, four outputs with 30–65% yields.

The synthesis of 4*H*-chromeno[4,3-*c*]isoxazol-4-one starting from 4-azido-3-hydroxycoumarin was scarcely reported. Vilsmeier–Haack reagent supplied the system with chloride and formyl moieties (**14**). The chloride was replaced by azido when compound **14** was treated by NaN_3_ to produce 4-azido-3-coumarin carboxaldehyde **15**_._ This last decomposed thermally to be depleted from nitrogen and spontaneously cyclized to 4*H*-chromeno[4,3-*c*]isoxazol-4-ones **103** (Scheme 32) [31].

### 2.2. Five-Membered Aromatic Rings with Three Heteroatoms

#### 2.2.1. Three Identical Heteroatoms

##### Triazole

**Figure 5:**
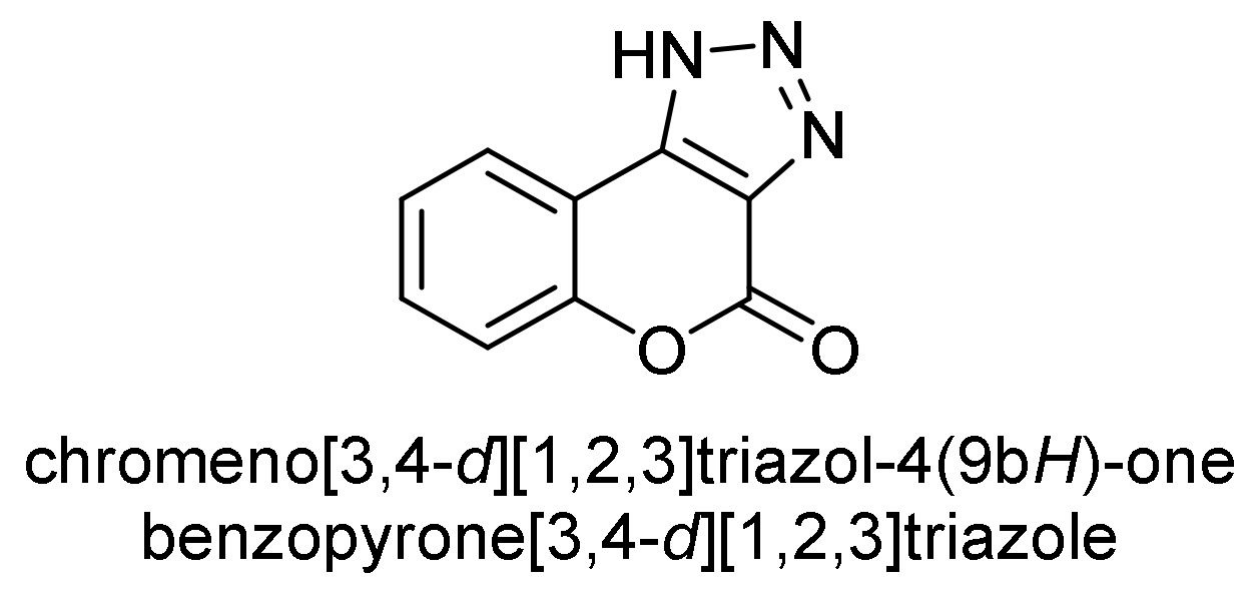
The common isomer of the fused chromeno-triazole system.

Fusion of the triazole ring with the pyrone ring of coumarin leads to one structural isomer (chromeno[3,4-d][1,2,3]triazol-4(9b*H*)-one) (Figure 5).

Chromeno[3,4-d][1,2,3]triazol-4(9b*H*)-one
Triazole Construction

**Scheme 33:**
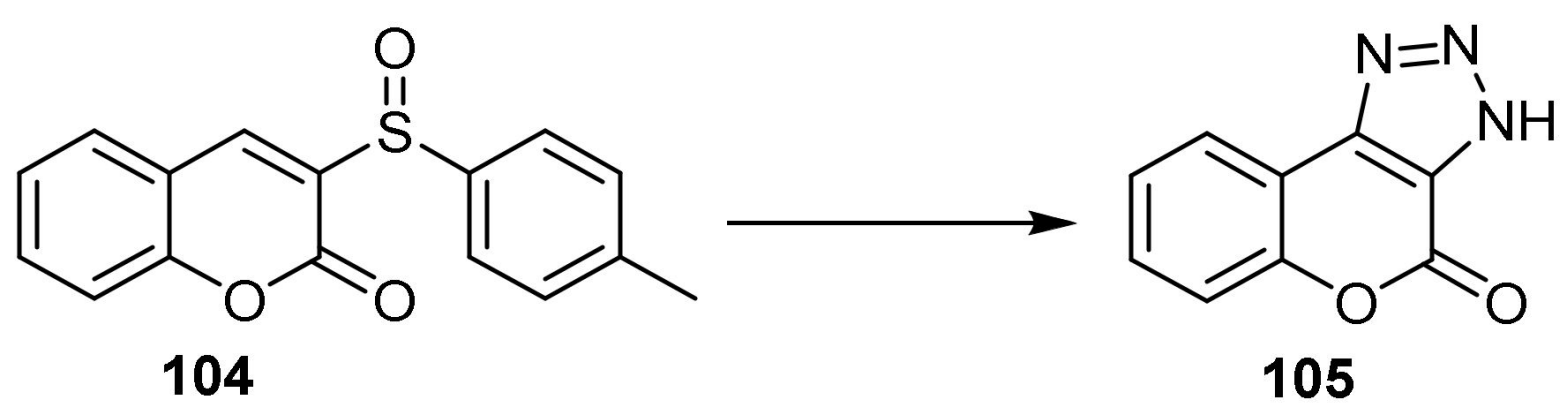
Synthesis of 4*H*-chromeno[3,4-d][1,2,3]triazol-(3H)4-one (**105**). Reagents and conditions. NaN_3_, DMF, 95 °C, 5 h, N_2_, 84% yield.

Dean and Park indicated the elimination of the sulphenic acid moiety during the addition of the corresponding 3-(4-methylphenylsulphinyl)coumarin (**104**) to sodium azide forming 4*H*-chromeno[3,4-d][1,2,3]triazol-(3H)4-one (**105**) (Scheme 33) [63].

**Scheme 34:**
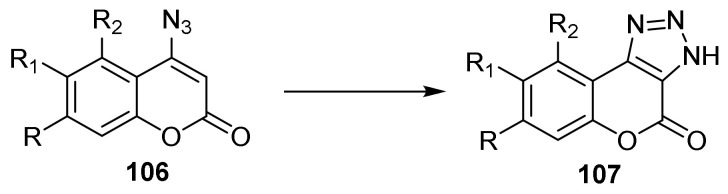
4-Azidocoumarins in the preparation of 4*H*-chromeno[3,4-d][1,2,3]triazol-(3H)4-ones **107**. Reagents and conditions. DMF or DMSO, *t*-BuOK, 50–60 °C, 5 h, stirring, six outputs with 12–41% yields.

A low to moderate yield of 4*H*-chromeno[3,4-d][1,2,3]triazol-(3*H*)4-ones **107** was obtained when 1,5-dipolar electro-cyclization took place within 4-azidocoumarins **106** that was induced by *t*-butoxide (Scheme 34) [64].

**Scheme 35:**
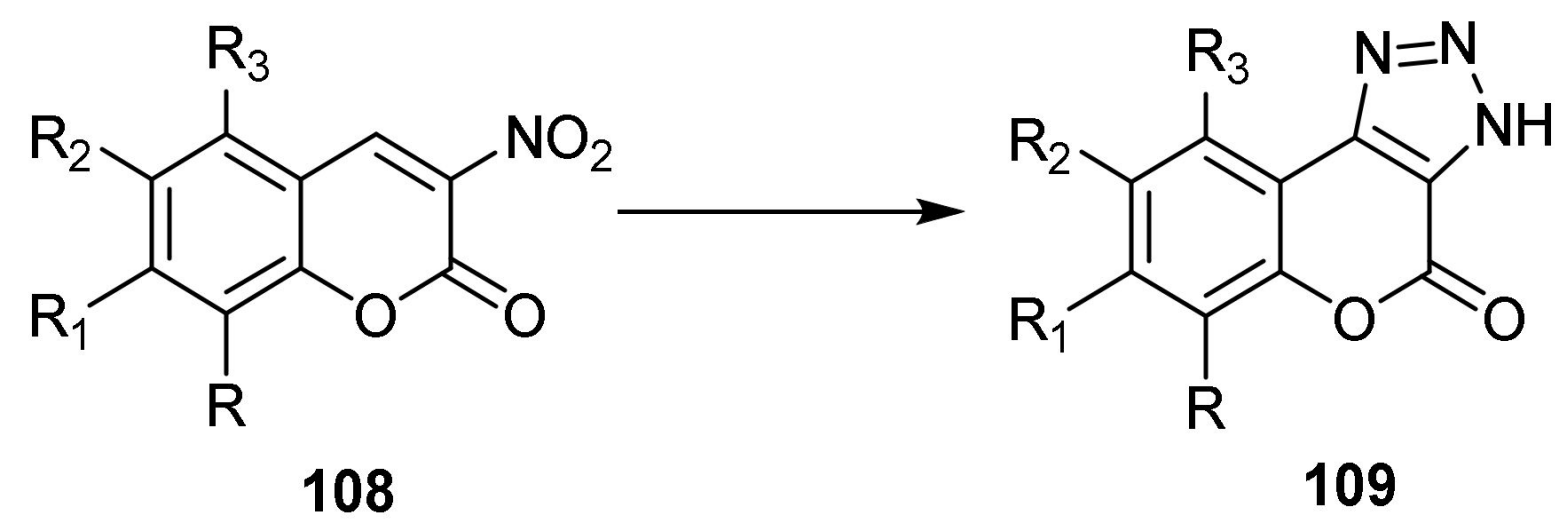
TBAF-catalyzed cycloadditions of 3-nitrocoumarins with TMSN_3_ under SFC. Regents and conditions. An amount of (10 mol%) TBAF, TMSN_3_ (2 equiv), 50 °C, eleven outputs with 76–94% yields.

A wide range of research demonstrated that 3-nitrocoumarin was the key compound for the synthesis of fused coumarin-triazole [65,66,67]. Vaccaro and his coworkers subjected the 3-nitrocoumarins **108** to a [3 + 2] cycloaddition with trimethylsilyl azide (TMSN_3_) under a solvent-free condition (SFC). Tetrabutylammonium fluoride (TBAF) acted as a catalyst during the reaction to supply a series of 4*H*-chromeno[3,4-d][1,2,3]triazol-(3H)4-ones **109** (Scheme 35) [65]. This method confirmed that ammonium halogen TBAF salt can be efficaciously employed as a non-metallic catalyst for activating the silicon–nitrogen bond under SFC.

**Scheme 36:**
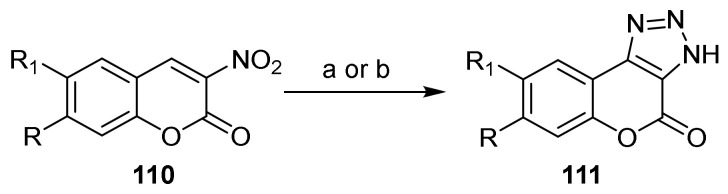
1,3-Dipolar cycloaddition of 3-nitrocoumarins with sodium azide. Reagents and conditions. a) NaN_3_, DMSO, 80 °C, 1 h, five outputs with 66–89% yields; b) NaN_3_ (1.2 equiv), DMF, Pyrex microwave vial equipped with a magnetic stir bar, stirred for 10 s, 160 C, 1 min, R = H, R_1_ = CH_3_ 94% yield; R = OCH_3_, R_1_ = H 69% yield.

In 2012, the formation of 4*H*-chromeno[3,4-d][1,2,3]triazol-(3*H*)4-ones **111** was described through a catalyst-free 1,3-dipolar cycloaddition of 3-nitrocoumarins **110** to sodium azide (Scheme 36). It was found that good yields were obtained in the presence of electron-withdrawing substituent on the aryl ring of 3-nitrocoumarins **110**. The reaction gave the best yield in DMSO at 80 °C after three lower temperatures attempts [66]. By applying this reaction, a novel group of 4H-chromeno[3,4-d][1,2,3]triazol-(3H)4-ones was achieved using microwave-assisted green chemistry procedures (Scheme 36) [67].

#### 2.2.2. Three Different Heteroatoms

##### Thiadiazole

**Figure 6:**
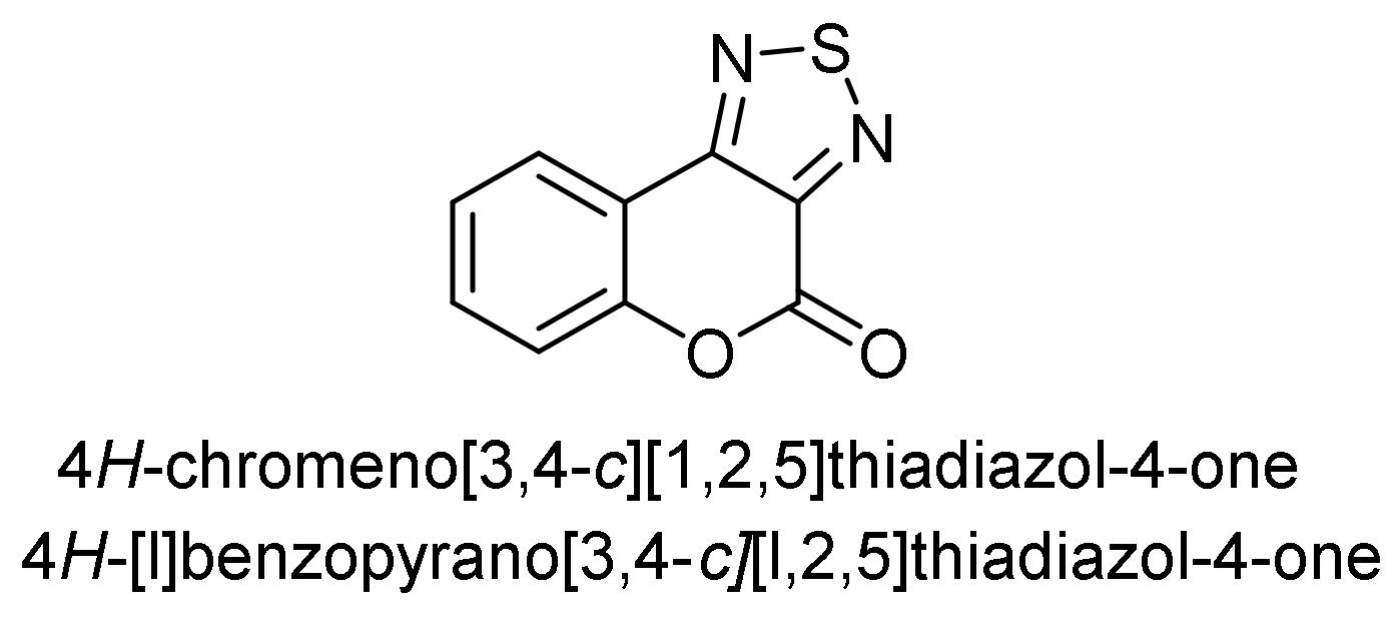
The common isomer of the fused chromeno-thiadiazole system.

Fusion of thiadiazole ring with the pyrone ring of coumarin furnishes one structural isomer, namely 4*H*-chromeno[3,4-c][1,2,5]thiadiazol-4-one (Figure 6).

4*H*-Chromeno[3,4-c][1,2,5]thiadiazol-4-one
Thiadiazole Construction

**Scheme 37:**
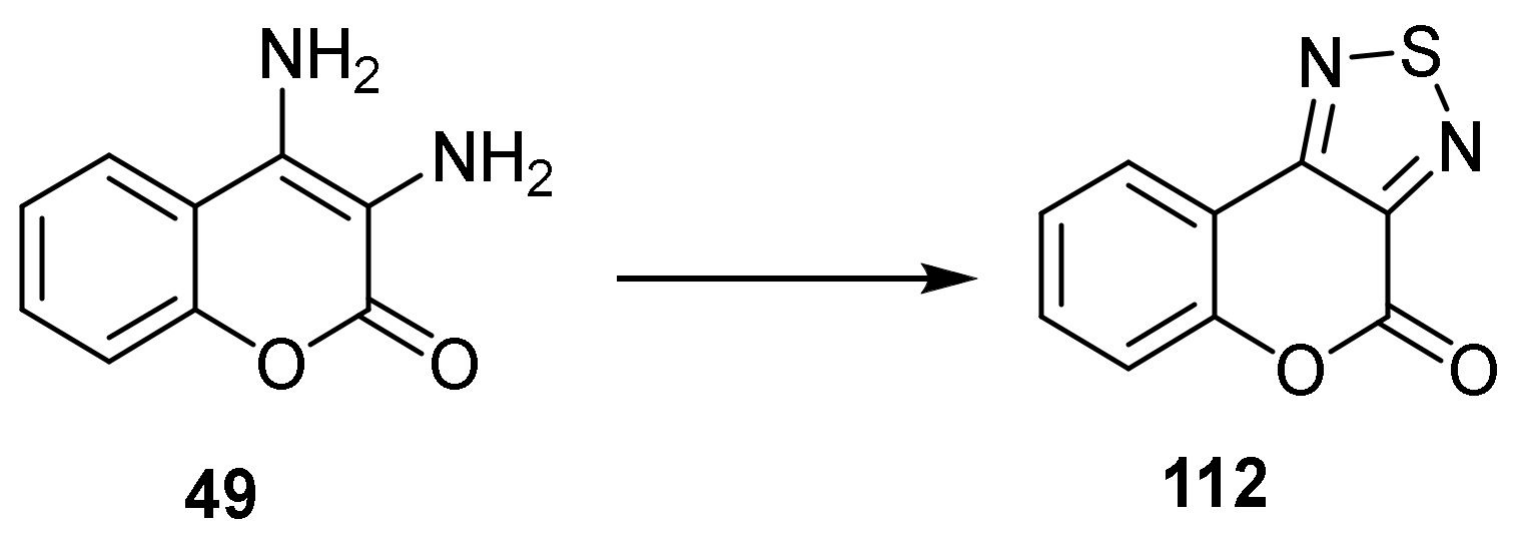
Synthetic pathway to 4*H*-chromeno[3,4-*c*][l,2,5]thiadiazol-4-one (**112**). Reagents and conditions. SOCl_2_, pyridine, stirring for 3 h, r.t., 87% yield.

Synthesis of the 4*H*-chromeno[3,4-c][1,2,5]thiadiazol-4-one was reported by only one research article that belongs to Smirnov and his co-workers [68]. The reaction of 3,4-diaminocoumarin (**49**) with thionyl chloride in pyridine gave 4*H*-chromeno[3,4-*c*][l, 2,5]thiadiazol-4-one (**112**) (Scheme 37).

**Figure 7:**
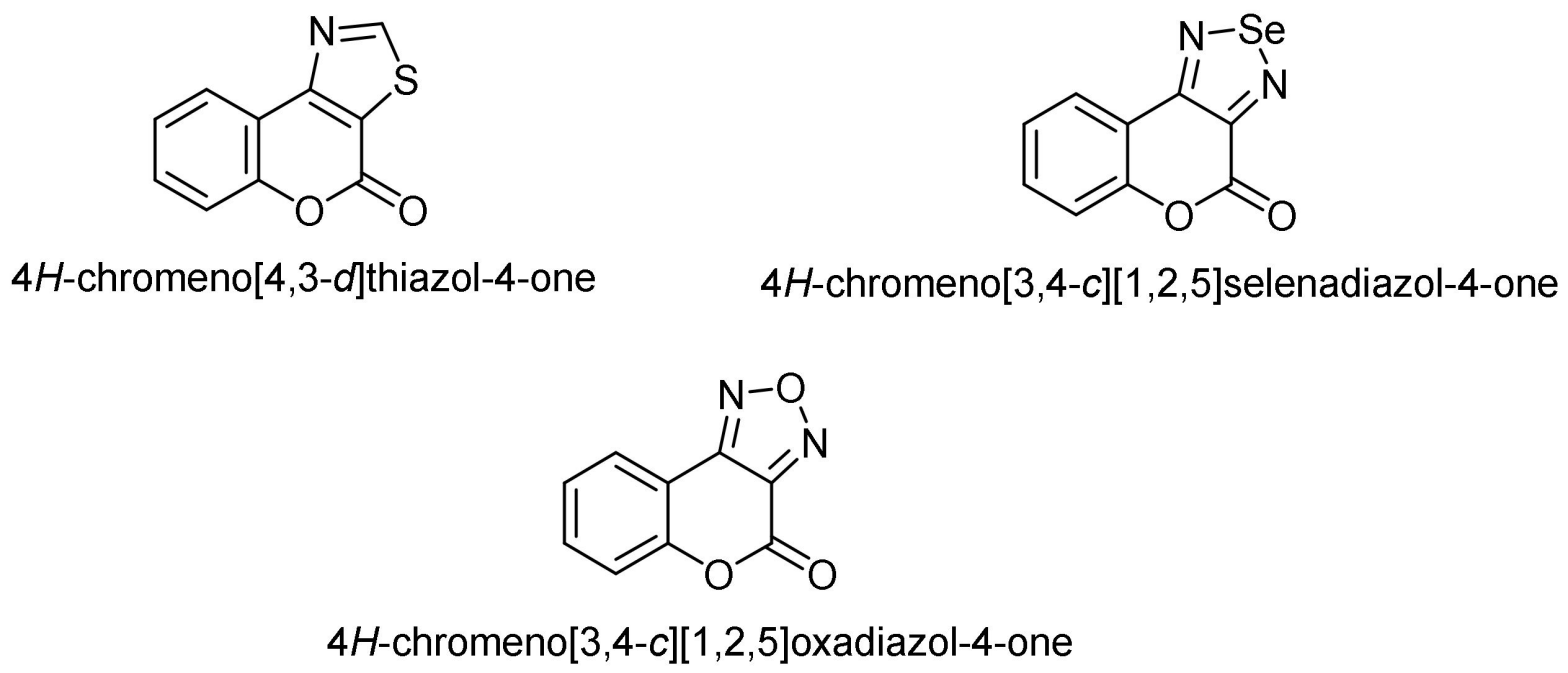
Coumarin-fused five-membered aromatic heterocycles have not been feasible by any synthetic procedures.

In summary, coumarins are one of the heterocyclic structures of great interest in the development of valuable biologically active structures. Since coumarins have versatile applications, synthesis trials of different structures of the coumarin-based scaffold were attempted. The different synthetic routes to synthesize coumarin (benzopyrone)-fused five-membered aromatic heterocycles with multi-heteroatoms built on the pyrone ring were discussed in this review to shed light on the evolution in synthetic methods. We found that the starting scaffolds for this preparation were mainly 4-hydroxy, 4-amino, 3,4-diamino, and 3-nitro derivatives of coumarin. Moreover, other various methods of building the pyrone ring from simple functionalized compounds were discussed. To date, 4*H*-chromeno[4,3-*d*]thiazol-4-one, 4*H*-chromeno[3,4-c][1,2,5]selenadiazol-4-one, and 4H-chromeno[3,4-c][1,2,5]oxadiazol-4-one (Figure 7) have not been feasible by any synthetic procedures.
